# Genetic engineering of grass cell wall polysaccharides for biorefining

**DOI:** 10.1111/pbi.12764

**Published:** 2017-06-30

**Authors:** Rakesh Bhatia, Joe A. Gallagher, Leonardo D. Gomez, Maurice Bosch

**Affiliations:** ^1^ Institute of Biological, Environmental and Rural Sciences (IBERS) Aberystwyth University Aberystwyth UK; ^2^ CNAP Department of Biology University of York Heslington York UK

**Keywords:** bioenergy, biomass, biotechnology, grasses, lignocellulose, transgenic plants

## Abstract

Grasses represent an abundant and widespread source of lignocellulosic biomass, which has yet to fulfil its potential as a feedstock for biorefining into renewable and sustainable biofuels and commodity chemicals. The inherent recalcitrance of lignocellulosic materials to deconstruction is the most crucial limitation for the commercial viability and economic feasibility of biomass biorefining. Over the last decade, the targeted genetic engineering of grasses has become more proficient, enabling rational approaches to modify lignocellulose with the aim of making it more amenable to bioconversion. In this review, we provide an overview of transgenic strategies and targets to tailor grass cell wall polysaccharides for biorefining applications. The bioengineering efforts and opportunities summarized here rely primarily on (A) reprogramming gene regulatory networks responsible for the biosynthesis of lignocellulose, (B) remodelling the chemical structure and substitution patterns of cell wall polysaccharides and (C) expressing lignocellulose degrading and/or modifying enzymes *in planta*. It is anticipated that outputs from the rational engineering of grass cell wall polysaccharides by such strategies could help in realizing an economically sustainable, grass‐derived lignocellulose processing industry.

## Introduction

Maize (*Zea mays*) and sugarcane (*Saccharum officinarum*) remain the world's largest biofuel‐producing feedstocks (Chum *et al*., 2014). These economic important grasses are currently utilized for respective starch and sucrose‐based bioethanol production via fermentation, and accounted for ~85 billion litres of bioethanol and ~85% of global bioethanol output in 2016 (Renewable Fuels Association, 2017). These ‘first‐generation’ biofuels offer in most cases an advantage in terms of carbon footprint compared to fossil fuels. However, with the increasing demand for agricultural land to satisfy the needs of a rapidly growing human population, alternative feedstocks for bioenergy and biorefining are required.

The utilization of abundant, diverse, carbon‐neutral, and non‐edible agricultural residues of grasses (*Poaceae*) including maize stover, sugarcane bagasse, rice and wheat straw, as well as the harvestable biomass of dedicated bioenergy crops including Miscanthus and switchgrass, represent crucial resources to realize the vision of a low‐carbon bioeconomy with biorefining into biofuels, platform chemicals, and value‐added bio‐based products at its core. The opening of several lignocellulosic‐based commercial‐scale biofuel plants (‘Beta Renewables’, ~50 million Litres of bioethanol per year (L/yr); ‘Project LIBERTY’, ~75 million L/yr; ‘DuPont’, ~110 million L/yr; ‘GranBio’, 82 million L/yr; ‘Raizen/Iogen’, 40 million L/yr) has been a landmark towards the establishment of commercially viable processes for ‘second‐generation’ biofuels. These new technology demonstrations will drive the demand for feedstocks that can fit the quality, as well as the scale required for these initiatives.

A number of crops have been explored as possible feedstock for biorefining, taking into account the carbon balance of using agricultural waste or selecting low‐input/high biomass yield species. Table 1 shows the agronomical and genetic features of the main grass lignocellulosic feedstocks explored to date. Corn stover, rice and wheat straw represent the most favourable agricultural wastes available as biomass resources (Table 1). Yet focus has generally been on the effective utilization of corn stover and wheat straw, with less consideration given to rice straw which is more abundant compared to the other major agricultural wastes (Table 1) (Binod *et al*., 2010; Sarkar *et al*., 2012). Until recently, rice straw was considered a waste stream of rice production with little or no value and farmers often burning it in the fields, causing health and environmental problems (Oanh *et al*., 2011). However, the potential of utilizing rice as a biorefining feedstock is increasingly being recognized (Abraham *et al*., 2016; Liu *et al*., 2016; Nguyen *et al*., 2016). Amongst the dedicated biomass crops with the highest potential for biorefining are the fast‐growing grasses, in particular, Miscanthus hybrids such as *Miscanthus × giganteus*, switchgrass (*Panicum virgatum*), and energy cane (a complex sugarcane hybrid with high lignocellulose yield) (Table 1). These C_4_ photosynthesizing grasses are principally coveted for their perenniality and high field productivity across temperature and drought environments, suitability for growth on marginal and erosive land, biodiversity promoting benefits, high water use efficiency and nutrient sequestering ability (Byrt *et al*., 2011; Carroll and Somerville, 2009; Clifton‐Brown *et al*., 2017; Feltus and Vandenbrink, 2012; Van der Weijde *et al*., 2013).

**Table 1:** Grass crops with high potential for genetic engineering and biorefining activities

Species	Mechanism of photosynthesis (carbon fixation)	Type	Average yield potential (dry tonne biomass/ha/yr)^a^	Genome sequencing status	Genome size (Mbp)	Genetic transformation system	References
Miscanthus (*Miscanthus *× *giganteus*)	C_4_	Crop	~22^b^	In progress	~7500	Not well established^g^	Swaminathan *et al*. (2010); Nordberg *et al*. (2014); Falter *et al*. (2015)
Sugarcane (*Saccharum officinarum*)	C_4_	Bagasse and field residue	~17^c^	In progress	~10 000	Established	Souza *et al*. (2011); De Setta *et al*. (2014); Dong *et al*. (2014); Mayavan *et al*. (2015); Wu and Altpeter (2015)
Energy cane (Saccharum complex hybrids)	C_4_	Bagasse and field residue	~50^d^	In progress (see sugarcane)	>10 000	Established^h^	Bischoff *et al*. (2008); Fouad *et al*. (2015); Leon *et al*. (2015); Anderson *et al*. (2016)
Sweet sorghum (*Sorghum bicolor*)	C_4_	Bagasse and field residue	~10^e^	Complete	~730	Established	Paterson *et al*. (2009); Raghuwanshi and Birch (2010); Liu and Godwin (2012)
Switchgrass (*Panicum virgatum*)	C_4_	Crop	~10^b^	In progress	~5600	Established	Xi *et al*. (2009); Ramamoorthy and Kumar (2012); Merrick and Fei (2015)
Rice (*Oryza sativa*)	C_3_	Straw	~6^f^	Complete	~390	Established	Sah *et al*. (2014)
Maize (*Zea mays*)	C_4_	Stover	~2^f^	Complete	~2400	Established	Klein *et al*. (1989); Huang and Wei (2005); Ishida *et al*. (2007); Frame *et al*. (2011); Que *et al*. (2014)
Wheat (*Triticum aestivum*)	C_3_	Straw	~2^f^	Complete	~16 500	Established	Li *et al*. (2012); Sparks *et al*. (2014)

Mbp, mega base pair.

a Yields are generally based on lignocellulosic biomass that can be harvested from fields without impacting soil fertility.

b Data was taken from Heaton *et al*. (2004).

c The global average dry bagasse yield was calculated as described by Van der Weijde *et al*. (2013), using the global average fresh sugarcane yield for 2014 (‘FAOSTAT’, 2016).

d Average dry yield based on total aboveground portion of the energy cane plant (stalks, tops, and leaves) taken from Anderson *et al*. (2016).

e Average dry sorghum bagasse and field residue yield was taken from Blümmel *et al*. (2009) and Van der Weijde *et al*. (2013).

f The global average rice, maize and wheat lignocellulosic yield was calculated using residue/crop ratios according to Kim and Dale (2004) and their respective average grain yields from 2014 (‘FAOSTAT’, 2016).

g Transformation not well established in *Miscanthus* ×* giganteus* except for a description in Falter *et al*. (2015) but established in *Miscanthus Sinensis* (Hwang *et al*., 2014; Wang *et al*., 2011).

h Transformation system established in Energy cane but with minimal transgene expression cassette (Fouad *et al*., 2015).

Lignocellulosic biomass accounts for ~60%–80% of dry matter yields in grasses and is primarily composed of secondary cell walls comprised mainly of cellulose (~25%–55%), hemicellulose (~20%–50%), and lignin (~10%–35%) (Marriott *et al*., 2015; Vogel, 2008). Secondary cell walls provide structural support, resist water loss, and protect against mechanical stress and breakdown by microbes. The complexity of the major structural and chemical components of secondary cell walls, which features a variety of chemical linkages within and between the main polymers, is the basis of lignocellulosic biomass recalcitrance and plays a key role in impeding the effective utilization of lignocellulose for bioconversion into fermentable sugars and value‐added products on an industrial scale. Efforts to make the deconstruction of lignocellulosic biomass economically viable and environmentally friendly have concentrated in three main areas: (i) improved pre‐processing (e.g. mechanical, thermochemical); (ii) improved processing through more efficient enzymes and microbes capable of tolerating toxic inhibitors, withstanding high product and by‐product concentrations during biomass digestion and the subsequent fermentation process, and (iii) developing less recalcitrant feedstocks (Agbor *et al*., 2011; Alvira *et al*., 2010; Balat, 2011; Klein‐Marcuschamer *et al*., 2012; Sarkar *et al*., 2012; Sims *et al*., 2010).

The key lignocellulose processing step in terms of energy and chemical demand is pretreatment, opening up the structure of the cell wall matrix, facilitating enzymes to access their substrates and improving hydrolysis of biomass polysaccharides (Galbe and Zacchi, 2012). Pretreatments modify the composition and architecture of the cell wall and can result in the production of fermentation inhibitors such as formic acid, acetic acid, or furfural, which often require removal prior to fermentation (Jönsson *et al*., 2013; Phitsuwan *et al*., 2013). While a wide range of pretreatments have been assessed, few have been implemented in commercial operations. These include the advanced steam explosion pretreatment technology by ANDRITZ Inc. and Proesa^®^ for Project LIBERTY and GranBio or Beta Renewables, respectively, the dilute acid pretreatment technology by Iogen for the Raizen project, and the more exploratory ones such as ionic liquids or the mild alkali pretreatment technology developed by the National Renewable Energy Laboratory for DuPont.

Lignocellulose depolymerisation enzyme discovery and improvement programmes have resulted in new generations of commercial enzyme cocktails that have improved the price competitiveness of cellulosic ethanol (Chandel *et al*., 2012). These programmes include: surveying enzymes produced by microbes isolated from a diverse range of environments including the rumen, compost heaps, hot springs and tropical forests as well as from ‘omic’ databases; modification of enzymes through computational biology and forced evolution; and genetic, metabolic and protein engineering techniques aimed at designing industrial microbial strains with proficient cellulolytic and hemicellulolytic activities (Banerjee, 2010).

Another option to increase the efficiency of lignocellulosic deconstruction and processing is the development of biomass tailored for these applications. Choices of feedstock species and breeding for less recalcitrant biomass while maintaining field performance including grain yield in dual‐purpose crops represent attractive approaches to improve process techno‐economics. Although breeding programmes on C_4_ grasses have been a time‐consuming and immensely complicated task due to screening of thousands of variants, chromosomal architecture, or multiplicity of alleles, the availability of modern genomic tools to deal with these complications opens the possibility of accurate mapping of genes and/or traits of interest that can be introduced in breeding strategies (Feltus and Vandenbrink, 2012; Slavov *et al*., 2013, 2014).

Alongside the progress in bioprocessing technologies, enzyme efficiencies, improved microbial strains, and feedstock choices, a complementary prospect to expedite biorefining of grass polysaccharides is via genetic engineering, which is the focus of this review. Although decoding the genetic and structural features that underpin cell wall recalcitrance remains complex, there has been a great deal of interest and progress in this area over the last 10 years. Here, we provide a brief overview of gene targets for genetic engineering of grass polysaccharides and highlight outcomes and perspectives of three different engineering strategies (A) reprogramming gene regulatory networks responsible for the biosynthesis of lignocellulose, (B) remodelling the chemical structure and substitution patterns of cell wall polysaccharides, and (C) expressing microbial lignocellulose degrading and/or modifying enzymes *in planta*. This review does not encompass all engineering efforts to date and does not focus directly on lignin modification or metabolism (covered elsewhere, (Furtado *et al*., 2014; Poovaiah *et al*., 2014; Cesarino *et al*., 2016)) due to the expanse of information on lignin biosynthesis genes and the effects of their manipulation on cell wall properties and digestibility (Eudes *et al*., 2014; Mottiar *et al*., 2016).

## The distinct features of grass cell walls

The cell walls of grasses consist of a complex composite framework composed mainly of polyphenol lignin (~10%–30%), cellulose (~35%–45%), and hemicellulose (~40%–50%) (for a review on lignocellulosic cell walls, their constituents and synthesis, see Marriott *et al*. (2015)). During the cell cycle in plants, dividing, expanding, or elongating cells have a distinctive primary cell wall. In the *Poaceae* family, the primary wall is thin, aqueous (~60%–70% water), and flexible, and is composed of ~1%–5% hydroxycinnamic acids (HCAs) such as ferulic acids (FA) and *p*‐coumaric acids (*p*‐CA), pectins (5%), and a few layers of cellulosic microfibrils (~20%–30%) embedded in a matrix of hemicelluloses such as mixed‐linkage glucans (MLGs) (~10%–30%) and highly substituted glucuronoarabinoxylans (GAXs) (~20%–40%) (O'Neill and York, 2003; Vogel, 2008). Upon cessation of cell enlargement, an additional and rigid secondary wall is deposited inside of the primary wall. This secondary cell wall, while containing negligible amounts of pectin (~0.1%), minor structural proteins and MLGs, HCAs (~0.5%–1.5%) and a small proportion of water (~5%), is primarily made up of hundreds of layers of cellulose microfibrils (~35%–45%) embedded in GAXs (~40%–50%) which in turn are covalently cross‐linked with hydrophobic polyphenol lignin (~20%) (Albersheim *et al*., 2011; Ebringerová *et al*., 2005; Vogel, 2008).

Depending on the tissue, cell type, cell wall layer, developmental stage, and plant taxa, the overall amount, architecture, and chemical composition of cell walls can vary significantly (Pauly and Keegstra, 2010). A characteristic feature of grass walls is the presence of particular polysaccharides such as GAX and MLG not found in the cell walls of woody biomass. Up to 40%–80% of the xylose residues of the xylan backbone can be substituted with *O*‐acetyl groups (Pauly *et al*., 2013). Another characteristic feature is the high amount of total FA (~4%) and *p*‐CA (~3%) as unbound acids or esterified to GAXs and ester‐ and ether‐linked to lignin in the primary and secondary walls of grasses, thereby cross‐linking these components (De Oliveira *et al*., 2015; Ishii, 1997; Lam *et al*., 2001; Ralph *et al*., 2004; Saulnier *et al*., 1999). Lignin is one of the main carbon components (~20%) of grass secondary walls and typically polymerized from three different 4‐hydroxyphenylpropanoids known as monolignols: *p*‐hydroxyphenyls (H) (~4%–15%), guaiacyl (G) (~35%–49%), and syringyl (S) (~40%–61%) (Boerjan *et al*., 2003). Such monolignols form diverse chemical bonds with each other at multiple positions (Boerjan *et al*., 2003), thereby crafting lignin as a heterogeneous aromatic and hydrophobic polymer that may lack a repeat structure. Hence, lignin tends to play a critical role in conferring cell wall rigidity and compactness by filling the voids between and around the cellulose and hemicellulose complexion, as well as fortifying the plant cell wall against biotic and abiotic responses. Collective evidence suggests that lignocellulosic biomass recalcitrance is dictated by several of the described cell wall components, their relative abundances, and interactions within the cell wall matrix.

**Figure 1:**
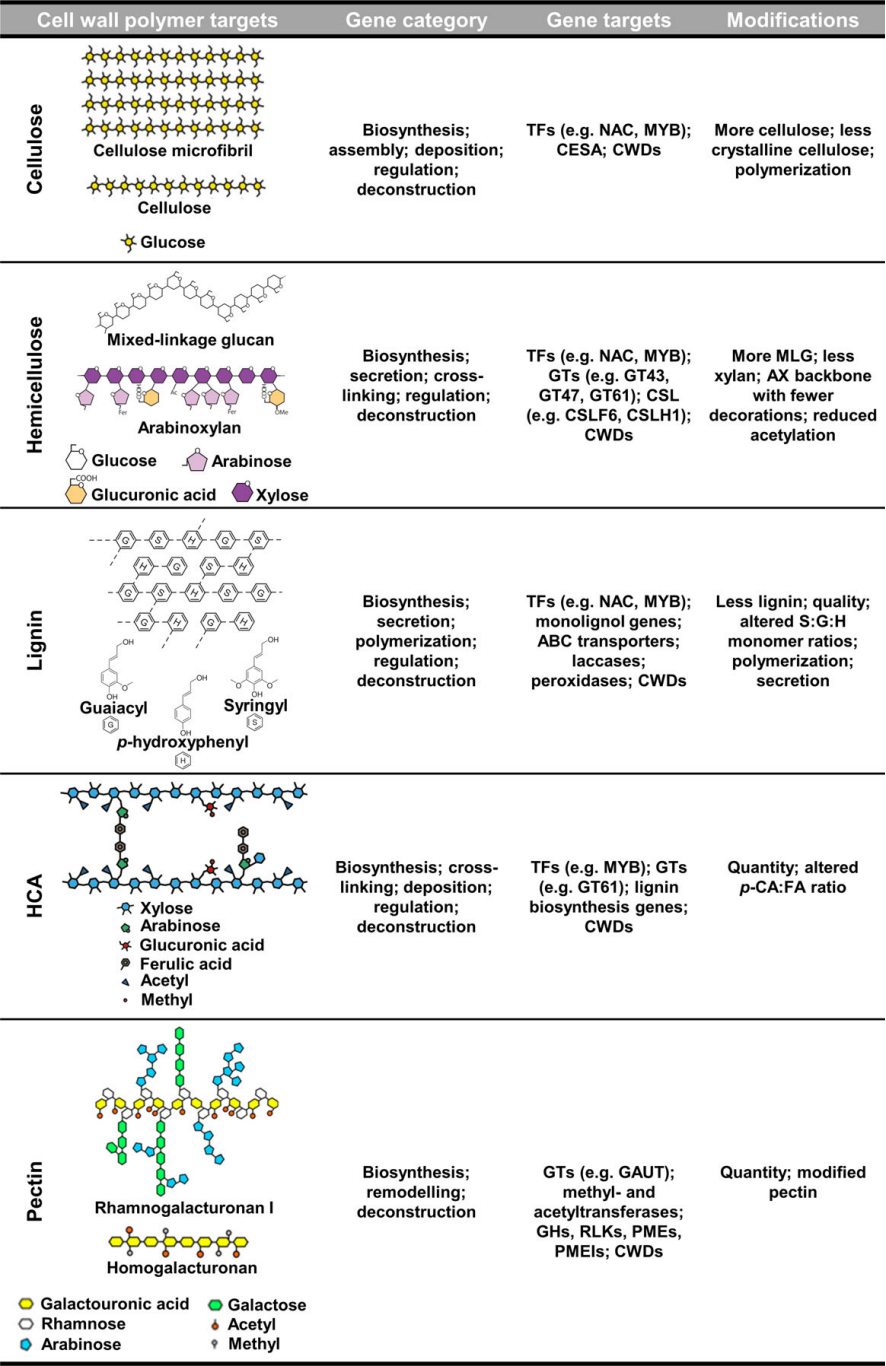
Cell wall polymer and associated gene targets for genetic engineering of grass biomass. Cell wall polymer targets were adapted from Rubin (2008), Harholt *et al*. (2010a), Scheller and Ulvskov (2010) and Marriott *et al*. (2015) and used with permission. ABC, ATP‐binding cassette transporters; AX, arabinoxylan; CESAs, cellulose synthase genes; CSL, cellulose synthase‐like gene; CWDs, cell wall degrading enzymes; FA, ferulic acid; G, guaiacyl units; GAUT, α‐(1,4) galacturonosyltransferase; GH, glycosylhydrolase; GT, glycosyltransferase; H, *p*‐hydroxyphenyl units; HCAs, hydroxycinnamic acids; MLG, mixed‐linkage glucan; MYB, Myeloblastosis; NAC, NAM,ATAF1,2 and CUC2; *p*‐CA,* p*‐coumaric acid; PME, pectin methylesterase; PMEI, pectin methylesterase inhibitor; RLK, receptor‐like kinase; S, syringyl units; TFs, transcription factors.

Efforts over the past decade have shown that engineering of grass cell walls using transgenic approaches can help overcome traits associated with cell wall recalcitrance. Researchers identified the need to select gene targets based on the different cell wall polymer targets they act upon, or different functionalities during cell wall construction or deconstruction, as categorized in Figure 1. These targets have driven most efforts to alter grass cell wall characteristics for effective downstream bioconversion, as reflected in the number of publications on this subject over the last decade (Tables 2, 3 and 4). We discuss the progress and perspectives of three different engineering strategies aimed at tailoring grass cell wall polysaccharides for biorefining applications.

**Table 2:** Literature related to transcriptional regulation of the cell wall by transcription factors

Transformed Gene	TF	ID	Source of transgene	Species	Transgenic approach	Promoter	Function/Results^a^	Plant phenotype^a^	References
*OsMYB46 ZmMYB46*	MYB	Os12g0515300/Os12g33070 JN634085	*Oryza sativa Zea mays*	*Arabidopsis thaliana*	Heterologous expression	35S	Activates cellulose, lignin, and xylan biosynthesis; induces ectopic deposition of lignin and xylan; increases cellulose accumulation	Strong curly leaves	Zhong *et al*. (2011)
*ZmMYB31*	MYB	GRMZM2G050305	*Zea mays*	*Arabidopsis thaliana*	Heterologous expression	35S	Directly represses lignin biosynthesis; decreases lignin content by 70%; 4‐fold increase in H monomer	Dwarfed with smaller leaf, stalk and flower size and delayed flowering	Fornalé *et al*. (2006, 2010)
*ZmMYB42*	MYB	GRMZM2G419239	*Zea mays*	*Arabidopsis thaliana*	Heterologous expression	35S	Represses lignin biosynthesis; decreases lignin content by 60%; 4‐fold increase in H monomer	Dwarfed with smaller leaves	Fornalé *et al*. (2006); Sonbol *et al*. (2009)
*ZmMYB31* *ZmMYB42*	MYB	GRMZM2G050305 GRMZM2G419239	*Zea mays*	*Saccharum spp. hybrids*	Overexpression	ZmUbi1	Represses lignin biosynthesis; decreases lignin content by ~13% in some lines; improves glucose release by ~30% in all *ZmMYB46* plants and by ~25% in two *ZmMYB31* plants	Little difference in plant height and number of internodes	Poovaiah *et al*. (2016)
*PvMYB4*	MYB	JF299185	*Panicum virgatum*	*Panicum virgatum*	Overexpression	ZmUbi1	Represses lignin biosynthesis; decreases lignin content by ~40%–50%; reduces *p*‐CA: FA ratio by ~50%; improves sugar release by ~3‐fold and ethanol yield by ~2.5‐fold	Reduced plant stature (~40%); increased tillering (~2.5‐fold)	Shen *et al*. (2012a, 2013)
*PvMYB46A*	MYB	AP13ISTG55477	*Panicum virgatum*	*Arabidopsis thaliana*	Heterologous expression	35S	Induces ectopic deposition of cellulose, lignin and xylan	Smaller rosette size, curly leaves	Zhong *et al*. (2015)
*SbMYB60*	MYB	Sb004G273800	*Sorghum bicolor*	*Sorghum bicolor*	Overexpression	35S	Increases lignin biosynthesis; 1.25–2.5‐fold increase in S monomer; ~2%–4% increase in energy content	Reduced plant height (~30%); delayed flowering	Scully *et al*. (2016)
*OsMYB103L*	MYB	Os08g05520	*Oryza sativa*	*Oryza sativa*	Overexpression RNA interference	ZmUbi1	*OsMYB103L* overexpression increases cellulose content by~13%; *OsMYB103L* RNAi decreases cellulose content by ~15%–30%	*OsMYB103L* overexpression causes inward rolled leaf; *OsMYB103L* RNAi reduces mechanical strength in leaves	Yang *et al*. (2014)
*TaMYB4*	MYB	JF746995	*Triticum aestivum*	*Nicotiana tabacum*	Heterologous expression	35S	Represses lignin biosynthesis; decreases lignin content by ˜16%–23%; increases S/G ratio by 36%–66% and leaf flavonoid content by 22%–29%	No morphological alterations except for dark green patches in leaves	Ma *et al*. (2011)
*OsSWN1* *OsSWN3* *OsSWN7* *ZmSWN1* *ZmSWN3* *ZmSWN7*	NAC	Os06g04090/Os06g0131700 Os08g01330/Os08g0103900 Os06g01480/Os06g0104200 JN634077 JN634079 JN634083	*Oryza sativa* *Zea mays*	*Arabidopsis thaliana*	Heterologous expression	35S	Activates cellulose, lignin, and xylan biosynthesis; induces ectopic deposition of cellulose, xylan and lignin	Strong curly leaves	(Zhong *et al*., 2011)
*OsSWN1* *OsSWN2S*	NAC	Os06g0131700 Os08g0115800	*Oryza sativa*	*Arabidopsis thaliana* *Oryza sativa*	Heterologous expression Chimeric repression	35S SRDX	Only *OsSWN1* heterologous expression induces secondary wall formation; *OsSWN2S* chimeric repression reduces wall thickening, lignin and xylose contents and increases digestibility by ~3%–4%	*OsSWN2S* chimeric repression results in drooping leaf phenotype	Yoshida *et al*. (2013)
*OsSWN1*	NAC	Os06g04090	*Oryza sativa*	*Oryza sativa*	Overexpression RNA interference	ZmUbi1	*OsSWN1* overexpression enhances lignin content by ~2‐6% and reduces saccharification yields by ~30%; *OsSWN1* silencing reduces lignin content by ~7%–20% and enhances saccharification yields by ~14%–43%	Most *OsSWN1* overexpression lines are semi‐dwarfed, sterile and have erect leaves; *OsSWN1* RNAi lines are normal but sterile	Chai *et al*. (2015)
*PvSWN1‐8*	NAC	KT075080‐93	*Panicum virgatum*	*Arabidopsis thaliana*	Heterologous expression	35S	Activates cellulose, lignin and xylan biosynthesis; induces ectopic deposition of cellulose, lignin, and xylan	Smaller rosette size; curly leaves	Zhong *et al*. (2015)
*BdSWN5*	NAC	JQ693422–JQ693429	*Brachypodium distachyon*	*Brachypodium distachyon*	Overexpression	Oestradiol‐inducible	Activates secondary wall gene synthesis and cell death	Normal	Valdivia *et al*. (2013)
*AtSHN2*	SHN	At5g11190	*Arabidopsis thaliana*	*Oryza sativa*	Heterologous expression	35S	34% increase in cellulose; 45% reduction in lignin	Normal	Ambavaram *et al*. (2011)
*PvERF001*	AP2/ERF	NR	*Panicum virgatum*	*Panicum virgatum*	Overexpression	ZmUbi1	Increases glucose release by ~10%–16%	~20%–100% increase in dry biomass yield	Wuddineh *et al*. (2015)

a May not encompass complete research findings.

ID, identifier; NR, not reported; RNAi, RNA interference; SRDX, EAR‐repression domain; ZmUbi1, maize ubiquitin 1 promoter; 35S, cauliflower mosaic virus promoter.

**Table 3:** Literature related to remodelling of grass cell wall polysaccharides

Transformed Gene	Annotation	ID	Source of transgene	Species	Transgenic approach	Promoter	Function/Results^a^	Plant phenotype^a^	References
*OsIRX9 OsIRX9L OsIRX14*	GT43	Os07g49370 Os01g48440 Os06g47340	*Oryza sativa*	*Arabidopsis thaliana*	Heterologous expression	35S	Increases xylan synthase activity	Restores *irx14* and *irx9* mutants	Chiniquy *et al*. (2013)
*OsGT43*	GT43	Os05g03174 Os05g48600 Os04g01280 Os06g47340	*Oryza sativa*	*Arabidopsis thaliana*	Heterologous expression	35S	Xylan backbone synthesis	Restores *irx14* and *irx9* mutants	Lee *et al*. (2014)
*MIGT43A‐G*	GT43	KX082754‐KX082760	*Miscanthus lutarioriparius*	*Arabidopsis thaliana*	Heterologous expression	35S	Xylan biosynthesis	*MIGT43A‐E* restores *irx9* mutant; *MIGT43F‐G* restores *irx14* mutant	Wang *et al*. (2016)
*TaGT43* *TaGT47*	GT43 GT47	HF913567‐9 HF913570‐2	*Triticum aestivum*	*Triticum aestivum*	RNA interference	HMW1Dx5	Decreases AX content by 40%–50%; increases degree of arabinosylation by 25%–30%; 50% decrease in cell wall thickness	Normal	Lovegrove *et al*. (2013)
*OsGT47A*	GT47	Os01g0926600/Os01g70190	*Oryza sativa*	*Arabidopsis thaliana*	Heterologous expression	35S	Restores secondary wall thickness and monosaccharide content	Restores plant growth in *irx10 irx10L* double mutant	Zhang *et al*. (2014)
*TaXAT1 TaXAT2 OsXAT3*	GT61	FR873610.1 FR846232.1	*Triticum aestivum* *Oryza sativa*	*Arabidopsis thaliana*	RNA interference	HMW1Dx5	Decreases Ara*f* substitution of xylan	Normal	Anders *et al*. (2012)
*OsUAM1*	UDP‐arabinopyranose mutase	Os03g0599800	*Oryza sativa*	*Oryza sativa*	RNA interference	ZmUbi1	Reduces arabinose by up to 44% and extent of xylan substitution; reduces FA and *p*‐CA contents by 25%–80%	Plants with >25% reduction in arabinose were dwarfed and infertile	Konishi *et al*. (2011)
*PvUAM1*	UDP‐arabinopyranose mutase	Pavirv000Ib03909	*Panicum virgatum*	*Panicum virgatum*	RNA interference	ZmUbi1	Reduces stem arabinose by up to 39%; increases level of stem cellulose by up to 38% and lignin by up to 13%; unchanged saccharification efficiency	Phenotypic differences between RNAi lines	Willis *et al*. (2016b)
*OsARAF1* *OsARAF3*	Arabinofuranosidase	Os07g0686900 Os11g0131900	*Oryza sativa*	*Oryza sativa*	Overexpression	ZmUbi1	~20%–25% decrease in arabinose content; ~28%–34% increase in glucose; ~46%–70% increase in saccharification	Normal	Sumiyoshi *et al*. (2013)
*OsCslF2* *OsCslF4* *OsCslF9*	Cellulose synthase	Os07g0552800 Os07g0553300 Os07g0551500	*Oryza sativa*	*Arabidopsis thaliana*	Heterologous expression	35S	Accumulation of MLG <0.1% of total wall	Normal	Burton *et al*. (2006)
*HvCesA4* *HvCesA8*	Cellulose synthase	HM222644 KM45970	*Hordeum vulgare*	*Hordeum vulgare*	Overexpression	35S	Reductions in cellulose content by 40%; decrease in cell wall thickness	Dwarfism; early‐stage leaf necrosis; stunted; brittle nodes	Tan *et al*. (2015)
*HvCSLH1*	Cellulose synthase	FJ459581	*Hordeum vulgare*	*Arabidopsis thaliana*	Heterologous expression	35S	Accumulation of MLG between 0.00015% and 0.016% of total wall	Normal	Doblin *et al*. (2009)
*HvCslF6*	Cellulose synthase	AB621333.1	*Hordeum vulgare*	*Hordeum vulgare*	Overexpression	35S	Up to 6‐fold higher MLG content in leaves	Often lethal; surviving plants have necrotic leaf tips	Burton *et al*. (2011)
*OsCslF6*	Cellulose synthase	Os08g0160500	*Oryza sativa*	*Arabidopsis thaliana*	Heterologous expression	SAG12	4‐times more glucose in the cell wall and ~42% increase in saccharification	Normal	Vega‐Sánchez *et al*. (2015)

a May not encompass complete research findings.

HMW1Dx5, starchy endosperm‐specific promoter; ID, identifier; SAG12, senescence‐associated gene 12; UDP, Uridine diphosphate; ZmUbi1, maize ubiquitin 1 promoter; 35S, cauliflower mosaic virus promoter.

**Table 4:** Literature related to genetic engineering of grass cell walls by *in planta* expression of cell wall degrading or modifying enzymes

Transformed Gene	Annotation	ID	Source of transgene	Species	Transgenic approach	Promoter	Function/Results^a^	Plant phenotype^a^	References
*EG* *CBH1*	Endoglucanase Cellobiohydrolase	E.C. 3.2.1.4 E.C. 3.2.1.91	*Acidothermus cellulolyticus* *Trichoderma reesei*	*Zea mays*	Heterologous expression	35S	Enzyme accumulated up to 2.1% TSP; enzymatic activity of 0.845 nmol/μg/min in leaf	Normal	Biswas *et al*. (2006)
*EG* *CBH1*	Endoglucanase Cellobiohydrolase	U33212 X69976	*Acidothermus cellulolyticus* *Trichoderma koningii*	*Zea mays*	Heterologous expression	Glob‐1	Enzyme accumulated >16% TSP	Normal	Hood *et al*. (2007)
*EG* *CBH1* *Bg1A*	Endoglucanase Cellobiohydrolase Cellobiase	E.C. 3.2.1.4 E.C. 3.2.1.91 E.C. 3.2.1.21	*Acidothermus cellulolyticus* Trichoderma reesei *Butyrivibrio fibrisolvens*	*Zea mays*	Heterologous expression	RbcS1 35S	Ratio of 1:4:1 (EG:CBH1:Bg1A) shows efficient conversion of pre‐treated corn stover	Normal	Park *et al*. (2011)
*CBH1* *CBH2* *EG*	Cellobiohydrolase Endoglucanase	FR719150 FR719151 FR719152	*Penicillium sp. Trichoderma sp*.	*Saccharum officinarum*	Heterologous expression	ZmPepC ZmUbi1	Endo‐ and Exoglucanase activity achieved in the leaves	Normal	Harrison *et al*. (2011, 2014b)
*CBH1*	Cellobiohydrolase	E.C. 3.2.1.91	*Penicillium sp*.	*Zea mays*	Heterologous expression	ZmPepC	Use of recombinant CBH1 enhanced performance of commercial cellulase mixture by up to 4‐fold on pre‐treated sugarcane bagasse	Normal	Harrison *et al*. (2014a)
*Cel‐Hyb1 (CelA and Cel6G)*	Endoglucanase	AY206451	*Neocallimastix patriciarum* *Piromyces sp*.	*Hordeum vulgare*	Heterologous expression	GluB‐1	Endoglucanase production of up to 1.5% of total grain protein remains stable post‐harvest	Normal	Xue *et al*. (2003)
*EG*	Endoglucanase	E.C. 3.2.1.4	*Acidothermus cellulolyticus*	*Oryza sativa*	Heterologous expression	35S	Enzyme accumulated up to 4.9% TSP; ~22%–30% of the cellulose converted into glucose	Normal	Oraby *et al*. (2007)
*EG*	Endoglucanase	E.C. 3.2.1.4	*Acidothermus cellulolyticus*	*Zea mays*	Heterologous expression	35S	Enzyme accumulated up to 1.13% TSP; Enhanced auto‐hydrolytic efficiency	Normal	Ransom *et al*. (2007)
*EG*	Endoglucanase	E.C. 3.2.1.4	*Acidothermus cellulolyticus*	*Zea mays*	Heterologous expression	RbcS1	Endoglucanase converts cellulose into fermentable glucose	Normal	Mei *et al*. (2009)
*AcCel5A*	Endoglucanase	E.C. 3.2.1.4	*Acidothermus cellulolyticus*	*Zea mays*	Heterologous expression	35S	Improves saccharification by 10%–15% after mild‐pretreatment	Normal	Brunecky *et al*. (2011)
*EG*	Endoglucanase	E.C. 3.2.1.4	*Acidothermus cellulolyticus*	*Oryza sativa*	Heterologous expression	Mac	Enzyme accumulated up to 6.1% TSP; enhances hydrolysis of cellulose to reducing sugars by 43%	Normal; high AcE1 expression reduces plant stature and delays flowering	Chou *et al*. (2011)
*EG*	Endoglucanase	E.C. 3.2.1.4	*Acidothermus cellulolyticus*	*Oryza sativa*	Heterologous expression	Gt1	Endoglucanase activity at ~830 U/g of dried seeds	Seeds smaller; plant dwarfing and early flowering	Zhang *et al*. (2012)
*Bgl7A*	Endoglucanase	EC 3.2.1.73	*Bispora sp. MEY‐1*	*Zea mays*	Heterologous expression	ZM‐leg1A	Endoglucanase activity at ~780 U/g of dried seeds	Normal	Zhang *et al*. (2013)
*EXG1* *ENG1* *BEG1*	Exoglucanase Endoglucanase Cellobiase	AK108835 AK102748 AK070962	*Oryza sativa*	*Oryza sativa*	Overexpression	ZmUbi1 Act1P	Enhances saccharification of transgenic *EXG1* rice stems by ~32%–58%; no activity detected for ENG1 and BEG1	Abnormalities in leaf and sterility; no transgenic *ENG1* plants regenerated; *BEG1* transgenic plants grow normal	Nigorikawa *et al*. (2012)
*EXG1*	Exoglucanase	AK108835	*Oryza sativa*	*Oryza sativa*	Overexpression	SGR	Enhances saccharification of transgenic *EXG1* rice by ~4‐8%	Normal	Furukawa *et al*. (2014)
*XynA*	Xylanase	E. C. 3.2.1.8	*Neocallimastix patriciarum*	*Hordeum vulgare*	Heterologous expression	GluB‐1 Hor2‐4	Xylanase remains stable post‐harvest	~90% fertile transgenic lines	Patel *et al*. (2000)
*XynA1*	Xylanase	E. C. 3.2.1.8	*Clostridium thermocellum*	*Oryza sativa*	Heterologous expression	35S	Xylanase activity at ~250 U/g detected in leaves and seed grains	Normal	Kimura *et al*. (2003)
*XynBM*	Xylanase	E.C. 3.2.1.8	*Clostridium stercorarium*	*Oryza sativa*	Heterologous expression	Act1	~80% xylanase activity maintained in leaves	Normal	Kimura *et al*. (2010)
*XynB* *BSX*	Xylanase	E.C. 3.2.1.8	*Clostridium stercorarium* *Bacillus sp*.	*Zea mays*	Heterologous expression	GluB‐4 rubi3	Enzyme accumulated up to 0.1% TSP; BSX and XynB accumulated up to 4.0% TSP and 16.4% TSP respectively in grains	Stunted plants; sterile grains	Gray *et al*. (2011)
*Xyn2*	Xylanase	E.C. 3.2.1.8	*Trichoderma reesei*	*Festuca arundinacea*	Heterologous expression	Act1 LmSee1	Modifies cell wall structure and reduces sugar release by 30%	Reduced plant growth; 10%–60% reduction in biomass accumulation	Buanafina *et al*. (2012)
*iXynB*	Xylanase	E.C. 3.2.1.8	*Dictyoglomus thermophilum*	*Zea mays*	Heterologous expression	NR	Improves glucose and xylose release by ~20%	Normal seeds and fertility	Shen *et al*. (2012b)
*ATX*	Xylanase	AY949 844 E.C. 3.2.1.8	*Thermobifida fusca*	*Oryza sativa*	Heterologous expression	35S	Xylanase activity at ~3 U/g in fresh leaves	Normal	Weng *et al*. (2013)
*AnAXE*	Xylan acetyltransferase	AN6093.2 EC 3.1.1.72	*Aspergillus nidulans*	*Brachypodium distachyon*	Heterologous expression	ZmUbi1	Reduces cell wall acetylation by 1.3‐fold	Normal	Pogorelko *et al*. (2013)
*XynA* *FAE*	Xylanase Ferulic acid esterase	NC000964.2 Y09330.2	*Bacillus subtilis* *Aspergillus niger*	*Triticum aestivum*	Heterologous expression	1DX5	8%–20% increase in AX content in all transformants; 10%–15% increase in arabinose to xylose ratio in *XynA* grain cell walls; 13%–34% decrease in FA content in *FAE* grain cell walls	Mostly sterile; transgenic offspring kernels are shrivelled	Harholt *et al*. (2010b)
*XynA* *XynB* *EG* *FAE*	Xylanase Endoglucanase Ferulic acid esterase	E.C. 3.2.1.8 E.C. 3.2.1.4 E.C. 3.1.1.73	*Dictyoglomus* *Thermophilum* *Thermomyces* *Lanuginosus* *Nasutitermes* *Takasagoensis* *Acidothermus* *Cellulolyticus* *Aspergillus niger*	*Zea mays*	Heterologous expression	NR	Plants expressing one or two CWD enzymes show improved sugar release; *EGA* and *EGA/XynA* plants show 55% improvement in ethanol production	NR	Zhang *et al*. (2011)
*FAE* *Xyn2*	Ferulic acid esterase Xylanase	E.C. 3.1.1.73 E.C. 3.2.1.8	*Aspergillus niger* *Trichoderma reesei*	*Festuca arundinacea*	Heterologous expression	Act1 LmSee1	Increases lignin by 23% and saccharification by 31%	Narrow and short leaves; ~70% decrease in biomass	Buanafina *et al*. (2015)
*FAE*	Ferulic acid esterase	E.C. 3.1.1.73	*Aspergillus niger*	*Festuca arundinacea*	Heterologous expression	Act1 LmSee1	Ferulic acid esterase activity at ~100–400 U/g in fresh leaves; reduces cell wall ferulates by ~14%–25%; increases *in vitro* dry matter digestibility by up to 4% in *FAE* plants with lower ferulate levels	Normal	Buanafina *et al*. (2010)
*FAE*	Ferulic acid esterase	E.C. 3.1.1.73	*Aspergillus niger*	*Festuca arundinacea*	Heterologous expression	Act1 ZmUbi1 35S HS LmSee1	Ferulic acid esterase activity at ~400‐500 U/g in fresh leaves with heat shock and senescence promoters respectively; increases *in vitro* dry matter digestibility by up to 14% in *FAE*‐Act1 plants	Normal	Buanafina *et al*. (2008)
*FAE*	Ferulic acid esterase	E.C. 3.1.1.73	*Aspergillus niger*	*Lolium multiflorum*	Heterologous expression	Act1	Ferulic acid esterase activity at ~25–400 U/g in fresh leaves; reduces cell wall ferulates by ~50%–85%; increases *in vitro* dry matter digestibility by up to 14%	Normal	Buanafina *et al*. (2006)
*AcPMEI*	Pectin methylesterase	E.C. 3.1.1.11	*Actinidia chinensis*	*Triticum durum cv. Svevo*	Overexpression	ZmUbi1	~2.5‐fold higher saccharification efficiency	Normal	Lionetti *et al*. (2010)
*Man5A*	Mannase	EC 3.2.1.78	*Bispora sp. MEY‐1*	*Zea mays*	Heterologous expression	ZM‐leg1A	Mannase activity at ~20–26 U/g of dried seeds	Lower plant height by ~3%	Xu *et al*. (2013)
*Aga‐F75*	Galactosidase	EC3.2.1.22	*Gibberella sp. strain F75*	*Zea mays*	Heterologous expression	ZM‐leg1A	Galactosidase activity at 10 U/g of dried seeds	Normal	Yang *et al*. (2015)
*OsEXP4*	Expansin	Os05g0477600	*Oryza sativa*	*Oryza sativa*	Overexpression and RNA interference	ZmUbi1	Expansin expression affects growth and development	Pleiotropic phenotypes in plant height, leaf number, flowering time, and seed set	Choi *et al*. (2003)
*OsEXPA8*	Expansin	Os01g0248900	*Oryza sativa*	*Oryza sativa*	Overexpression	35S	Enhances cell size of leaf and root vascular bundles in transgenic rice	Increased plant height (~10%), leaf size (~16%) and root length (~36%)	Ma *et al*. (2013)
*OsEXPA8*	Expansin	Os01g0248900	*Oryza sativa*	*Oryza sativa*	RNA interference	ZmUbi1	Reduces cell size; increases cell wall stiffness; inhibits cell growth	Reduction in plant height and growth	Wang *et al*. (2014)

a May not encompass complete research findings.

Act1, rice actin 1 promoter; Glob‐1, maize embryo‐preferred globulin‐1 promoter; GluB‐1, barley glutelin B‐1 promoter; GluB‐4, rice glutelin 4 promoter; Gt1, rice glutelin 1 promoter; Hor2‐4, hordein gene promoter; HS, soybean heat shock promoter; ID, identifier; LmSee1, Lolium multiflorum senescence‐enhanced gene promoter; Mac, a hybrid promoter of mannopine synthase promoter and cauliflower mosaic virus 35S promoter enhancer region; NR, not reported; RbcS1, rubisco small subunit promoter; rubi3, rice ubiquitin 3 promoter; SGR, stay green promoter; TSP, total soluble plant protein; ZM‐leg1A, maize legumin 1A (leg1) promoter; ZmpepC, maize phosphoenolpyruvate carboxylase promoter; ZmUbi1, maize ubiquitin 1 promoter; 1DX5, endosperm‐specific 1DX5 glutenin promoter; 35S, cauliflower mosaic virus promoter.

## A. Reprogramming grass cell wall gene regulatory networks

There are several major plant transcription factor (TF) families, including basic Helix‐Loop‐Helix (bHLH), Homeobox (HB), basic‐region leucine zipper (bZIP), Auxin/indole‐3‐acetic acid (AUX/IAA) and APETALA2/Ethylene Responsive Factor (AP2/ERF), potentially implicated in regulating secondary cell wall biosynthesis (Cassan‐Wang *et al*., 2013; Hirano *et al*., 2013). Within the secondary cell wall TF network, two favourable targets for grass cell wall engineering have been the R2R3‐MYB (MYELOBLASTOSIS) and NAC (NAM, ATAF, CUC) TF family members (Table 2). These proteins form one of the largest plant‐specific TF families and play a key role in regulating cell wall formation (Dubos *et al*., 2010; Olsen *et al*., 2005). Hence, modified expression of *MYB* and *NAC* TF genes are expected to reprogram cell wall biosynthesis, providing a route towards improving relevant grass cell wall traits (Bhatia and Bosch, 2014). TFs are sequence‐specific DNA binding proteins that *trans‐*modify the transcription of target genes quantitatively, temporally (developmental stage‐specific), spatially (tissue‐specific) or in a stimulus‐dependent manner. Thus, understanding the biological role of TFs is important to fully harness their potential as a genetic tool for the improvement of grass wall characteristics. Research efforts have revealed an extensive, complex, hierarchical, and multilevel regulatory network of *MYB* and *NAC* TF genes in the dicot model species *Arabidopsis* (Hussey *et al*., 2013; Taylor‐Teeples *et al*., 2015). Although some grass MYB and NAC TFs have been shown to regulate secondary cell wall biosynthesis (Fornalé *et al*., 2010; Sonbol *et al*., 2009; Valdivia *et al*., 2013; Zhong *et al*., 2011), the model of the grass cell wall transcriptional regulatory network is still not as well defined (Handakumbura and Hazen, 2012).

There have been relatively few but valuable attempts in the reprogramming of grass cell wall gene regulatory networks (GRNs) by transgenic approaches (Table 2). For instance, overexpression (OX) of *PvMYB4* in switchgrass not only reduced lignin content and ester‐linked *p*‐CA:FA ratio by ~50%, but also improved cellulosic ethanol yield by ~2.5‐fold (Shen *et al*., 2012a, 2013). Conversely, overexpression of *SbMYB60* in sorghum was associated with increased lignin biosynthesis, resulting in a higher energy content of the biomass (Scully *et al*., 2016). However, both overexpression of *PvMYB4* and *SbMYB60* altered several plant growth characteristics, including a significant reduction in plant height (~40% and ~30% respectively). These findings suggest that there is a limit in the plasticity of grasses to tolerate TF‐based manipulations in biomass composition without significant impediments in cell wall expansion during plant growth and development. Overexpression of *PvKN1* (Knotted1‐like) and *PvERF001* (AP2/ERF) TF genes in switchgrass enhanced saccharification (Wuddineh *et al*., 2015, 2016), with the former altering the expression of lignin, cellulose and hemicellulose biosynthetic genes, as well as the gibberellin biosynthesis pathway (Wuddineh *et al*., 2016), while no significant changes in lignin content and composition were detected for the latter (Wuddineh *et al*., 2015). However, as before, transgenic plants exhibited altered growth phenotypes, with *PvKN1*‐OX lines often showing inhibited shoot and root elongation while *PvERF001*‐OX lines showed a ~20%–100% increase in dry biomass yield. Despite the apparent absence of a direct association with cell wall regulatory and biosynthetic pathways, the *PvERF001*‐OX study shows that TFs can simultaneously improve enzymatic saccharification and biomass yield. Interestingly, transgenic sugarcane overexpressing the maize transcription factor *ZmMYB42* showed a significant reduction in lignin content (8%–21%) and released ~30% more glucose with minimal phenotypic effects (Poovaiah *et al*., 2016). Besides highlighting the potential of using TFs to increase sugar release by a modest reduction in lignin content, this study also emphasized the difficulties in predicting outcomes of modifying gene expression levels, particularly in grasses with large complex polyploid genomes, and the need to better understand metabolic fluxes through the cell wall biosynthesis pathways.

Some of our knowledge of grass‐specific secondary cell wall‐related TFs comes from the study of the rice TFs, *OsMYB103L* and *OsSWN1*, which were characterized by overexpression and RNA interference (RNAi) techniques (Chai *et al*., 2015; Yang *et al*., 2014) (Table 2). The expression levels of several cellulose synthases (*CesAs*) in *OsMYB103L*‐OX lines were significantly increased along with cellulose content (~13%). Concordantly, RNAi of *OsMYB103L* led to a reduction in cellulose content (~15%–30%) and expression levels of *CESA* genes as well as impaired mechanical strength in leaves (Yang *et al*., 2014), common phenotypes associated with *CESA* mutants such as *brittle culm13* (*bc13*) in rice and *irregular xylem* (*irx1* to *irx3*) in *Arabidopsis* (Song *et al*., 2013; Tanaka *et al*., 2003; Turner and Somerville, 1997). Overexpression of the NAC TF *OsSWN1* increased lignin content by ~2%–6% and decreased the glucose yield by ~30%, while RNAi lines showed a concomitant decrease in lignin content by ~7%–20% and increase in glucose yield by ~14%–43% (Chai *et al*., 2015). Both OX and RNAi lines showed abnormal developmental phenotypes with most *OsSWN1*‐OX lines displaying a semi‐dwarfed and nearly sterile phenotype, while RNAi lines had a relative normal growth phenotype but were sterile.

It is evident that manipulation of cell wall composition and sugar release by altering the expression of certain TFs is often accompanied by aberrant plant growth and fitness penalties (Table 2). Such phenotypic effects can either be a direct result of TF‐induced changes in cell wall composition or due to pleiotropic effects as a cell wall‐associated TF may also be involved in the regulation of developmental processes or in the response to biotic and abiotic stresses (Fornalé *et al*., 2010; Zhong *et al*., 2010). Overexpression studies can also lead to metabolic spillover into related pathways, and TFs may lose some target specificity when expressed at high levels (Martin *et al*., 2012). Such off‐target effects may make TFs perhaps less tractable and more challenging as tools for grass cell wall engineering. In this context, TF‐based genetic engineering studies require additional supporting data for interpretations. Only a limited number of studies have deepened into the evidence behind gene targets and protein–protein interactions of grass‐specific TFs involved in secondary cell wall transcriptional regulation. Shen *et al*. (2012a) for instance, identified *cis*‐regulatory elements (i.e. TF‐binding motifs) such as AC‐rich elements of monolignol pathway genes recognized by *PvMYB4*. Chromatin immunoprecipitation (ChIP) followed by microarray (ChIP‐chip) or sequencing (ChIP‐seq) could be key techniques to uncover direct or indirect target genes and binding sites of TFs (Agarwal *et al*., 2016; Zhu *et al*., 2012) to increase our understanding of the network dynamics and functionality for secondary wall formation. Additionally, yeast one‐hybrid (Y1H) assays represent powerful complements to ChIP for identifying and constructing transcriptional GRNs (Kim *et al*., 2013; Zhang *et al*., 2016), though Y1H assays have their own set of limitations (Reece‐Hoyes and Walhout, 2012). For a summary of the pros and cons of TF‐based genetic engineering and advantages and challenges of the methodologies used to infer transcriptional regulatory networks, see Zhang, 2003; Broun, 2004; Grotewold, 2008 and Hussey *et al*., 2013.

Much of the initial work on the transcriptional regulation of secondary wall biosynthesis has been based on *Arabidopsis*, with ~45% of the systematic analysis of grass TFs conducted using heterologous studies in transgenic *Arabidopsis* (Table 2). Given the relatively large genome size and TFs family divergence in grass species (Du *et al*., 2012; Pereira‐Santana *et al*., 2015), it remains questionable whether cell wall biosynthesis GRNs are equally conserved and wholly generalizable amongst dicot and monocot plant species. For example, while MYB58 and MYB63 act as lignin‐specific transcriptional activators in *Arabidopsis* (Zhou *et al*., 2009), the putative rice *(Oryza sativa)* orthologue OsMYB58/63 also regulates cellulose biosynthesis (Noda *et al*., 2015). Promoter analysis suggested that differences and similarities in the transcriptional regulation of lignocellulose biosynthesis genes between rice and *Arabidopsis* may be due to the distinct *cis*‐element composition of their promoters (Noda *et al*., 2015). This highlights the importance of characterizing TFs regulating secondary cell wall biosynthesis in grasses as the functionality of such TFs cannot be derived solely from functions defined by their dicotyledonous orthologs. The two genetic grass model systems *Brachypodium distachyon* and *Setaria viridis* could be alternative complementary resources to mine and validate genes and GRNs for grasses (Brutnell *et al*., 2015). Moreover, reprogramming approaches of grass cell wall GRNs have so far mostly been crude with not much variety in the selection of promoters for TFs to modify transcription of downstream target genes temporally, spatially or in a stimulus‐dependent manner (Table 2). Therefore, despite the potential of TF‐based genetic engineering strategies to reprogram grass cell wall GRNs, ample work is still necessary to fully dissect the roles of grass‐specific TFs in cell wall biosynthesis and to eliminate or at least mitigate against possible plant phenotype drawbacks.

## B. Remodelling grass cell wall polysaccharides

### Cellulose

Cellulose is the main component of plant lignocellulosic biomass and the most abundant terrestrial source of carbon. As a tightly packed microfibril of linear chains of β‐(1,4)‐linked glucose residues predominantly composed of crystalline domains that exhibit strong intra‐ and inter‐molecular bonding, cellulose has remarkable structural properties with a tensile strength equivalent to that of steel (Cosgrove, 1997). The strong inter‐chain hydrogen bonding network that gives cellulose its sturdy structural properties also makes it resistant to enzymatic hydrolysis, with an inverse correlation between cellulose crystallinity and the initial rate of cellulose hydrolysis (Hall *et al*., 2010). Hence, engineering approaches rendering crystalline cellulose more amorphous are a major research focus (for a comprehensive review see: Abramson *et al*., 2010). Initial studies, however, showed that such a target compromised other important plant agronomic traits. Harris *et al*. (2012) showed that in *Arabidopsis* two CESA mutants reduced the crystallinity of the cellulose microfibrils compared to the wild type. Lignocellulosic extracts of these mutants showed less recalcitrance in saccharification assays (49% increase in sugar release for the double mutant). However, the mutants, in particular the double mutant, exhibited dwarfed phenotypes. To this end, it seems that the targeted expression of exogenous cell wall degrading or modifying enzymes, explained in more detail in Section C, could provide a better route to alter cellulose crystallinity without compromising plant performance (Table 4).

Another biotechnological target has been to increase the amount of cellulose per unit of biomass, increasing the ratio of more easily fermented glucose monosaccharides (hexoses) compared to pentoses (mainly xylose derived from xylans). As cellulose is synthesized by hexameric rosette CESA complexes located at the plasma membrane (Carpita, 2012), increasing the amount and activity of grass‐specific CESA's, such as of OsCESA4, 7 and 9 that form the CESA complex typical for secondary cell wall biosynthesis in rice (Tanaka *et al*., 2003), appears as a logical approach. Attempts to implement such a strategy in barley (*Hordeum vulgare*) resulted in pleiotropic phenotypes and transcript silencing (Tan *et al*., 2015). An alternative approach would be to specifically target the transcriptional regulation of secondary cell wall cellulose synthases. This could theoretically lead to variations in cellulose synthesis with consequences on the orientation/organisation of cellulose microfibrils, possibly improving biorefining capabilities. However, there are no reports on the existence of such TFs. Overall, it remains questionable if reducing cellulose crystallinity and increasing cellulose abundance in grasses by altering the expression of endogenous genes can be achieved without a significant penalty on plant growth and performance.

### Xylan

The major grass hemicellulose sugar, xylan, varies in the number of substituents and side chains but is predominantly composed of a linear backbone of β‐(1,4)‐linked xylose residues often substituted with single residues of α‐(1,2)‐linked glucuronic acid (GlcA)/4‐*O*‐methylglucuronic acid (MeGlcA), α‐(1,2)‐ and/or α‐(1,3)‐linked arabinofuranosyl (Ara*f*), as well as less frequent disaccharide side chains including α‐(1,3)‐linked Ara*f* substituted with α‐(1,3)‐linked Ara*f* or β‐(1,2)‐linked xylose (Ebringerová and Heinze, 2000). In addition to sugar substitutions, xylosyl residues of xylan may also be *O*‐acetylated, and Ara*f* residues on the xylan backbone may be esterified with FA or *p*‐CA, the former covalently cross‐linking with lignin or adjacent xylan chains to strengthen secondary walls (Faik, 2010) (for a review on the detailed structure of hemicelluloses, see Scheller and Ulvskov (2010); for a xylan biosynthesis review, see Rennie and Scheller (2014)). This diverse pattern of possible xylan substitutions affects xylan conformation and solubility, and consequently grass cell wall architecture, all key determinants of saccharification yields. It also has implications regarding the need for complex enzyme mixtures to completely hydrolyse this polysaccharide to fermentable sugars.

Xylan acetylation is one of the main factors determining the insolubility and assembly of the xylans *in muro*. Deacetylation of maize stover by dilute alkaline extraction improves xylose monomer yields by ~10% upon pretreatment (Chen *et al*., 2012). The same study also showed that deacetylation of maize stover prior to dilute acid pretreatment results in ~20% higher saccharification yield compared to the same material acid pre‐treated. Studies in *Arabidopsis* likewise showed *O*‐acetylation levels to affect the physicochemical properties of xylan, plant growth and the enzymatic degradation of wall polymers (Schultink *et al*., 2015; Yuan *et al*., 2016). The presence of acetyl groups not only appears to be an impediment to enzymatic degradation but the release of acetate, mainly derived from deacetylation of xylan and pectins, may also act as yeast fermentation and enzyme digestion inhibitors (Helle *et al*., 2003; Pawar *et al*., 2016; Selig *et al*., 2009). Genes involved in xylan acetylation have not yet been characterized in grasses and understanding the mechanisms of polysaccharide *O*‐acetylation or modulating acetyltransferase activities might provide routes to enhance the conversion efficiency of lignocellulosic grasses to biorefining.

Given the diverse structural features of xylan, multiple modifying enzymes such as acetyltransferases and methyltransferases along with at least five glycosyltransferase (GT) enzyme activities, namely β‐(1,4) xylan synthase, α‐(1,2) glucuronyltransferase (GlcAT), α‐(1,2) or α‐(1,3) arabinofuranose transferase (AraT) and β‐(1,2) xylosyltransferase (XylT), are assumed to be involved in the xylan biosynthetic mechanism within the Golgi apparatus (Faik, 2010). Concurrently, these enzymes represent added targets and hold promise for engineering grass cell wall xylan. The importance of xylan side branches in changing the accessibility of lignocellulolytic enzymes is demonstrated by the dramatic effect of arabinofuranosidase (*OsARAF*) overexpression in rice, where the arabinose content decreased by 20%–25% while the glucose content increased by ~28%–34%, resulting in ~46%–70% improvement in saccharification efficiency with no visible phenotype (Sumiyoshi *et al*., 2013). Another report explored the significance of xylan backbone substitutions in transgenic rice via RNAi to suppress uridine diphosphate (UDP)‐arabinopyranose mutase (*OsUAM1*) expression, an enzyme that catalyses the formation of UDP‐Ara*f* from UDP‐arabinopyranose (UDP‐Ara*p*) (Konishi *et al*., *2011*). Although a reduction of 6%–44% in Ara*f* as well as 25%–80% reductions in the FA and *p*‐CA contents of the cell wall was observed, those transgenic rice plants with a >25% reduction in Ara*f* content were dwarfed and infertile (Konishi *et al*., *2011*). UAM's potential role in the recalcitrance of grass cell walls was recently investigated using RNAi to down‐regulate the expression of *PvUAM1* in switchgrass (Willis *et al*., 2016b). While there was an up to 39% decrease in cell wall‐associated arabinose from stem, a concurrent increase in cellulose (up to 38%) and lignin (up to 13%) content was observed in stems of *PvUAM*‐RNAi transgenic lines. This potential compensation response to maintain cell wall integrity may be the reason why enzymatic saccharification efficiency was unchanged (Willis *et al*., 2016b). However, it must be noted that reducing the number of xylan side chains with the aim of reducing wall cross‐linking and recalcitrance might also lead to structural changes and perhaps a denser cell wall matrix. Indeed, removal of arabinofuranose side chains decreased arabinoxylan (AX) solubility (Anders *et al*., 2012), possibly induced by increased hydrogen bonding between neighbouring AX chains.

A role in xylan biosynthesis for rice and Miscanthus GTs, mainly belonging to the GT43 and GT47 families, has been confirmed by their overexpression in *Arabidopsis irx* mutants. The complementation of the mutant phenotypes verified the function of each GT (Table 3). Other candidate genes with the same function in grasses have also been identified and characterized. For example, in wheat, the *IRX9* homologue *TaGT43_2* and the *IRX10* homologue *TaGT47_2* have been implicated in the biosynthesis of AX (Lovegrove *et al*., 2013). Additionally, two maize GT47 genes (*GRMZM2G100143* and *GRMZM2G059825*) identified via differential gene expression profiling in internodes undergoing secondary wall deposition represent likely candidates for involvement in the biosynthetic process of grass cell wall xylan (Bosch *et al*., 2011). Although modification of cell wall xylan content, composition and assembly/cross‐linking have been explored using grass‐specific and Golgi‐localized GT enzymes, less attention has been paid to enzymatic saccharification benefits that could arise from such transgenic modifications (Anders *et al*., 2012; Chiniquy *et al*., 2013; Lee *et al*., 2014; Lovegrove *et al*., 2013; Zhang *et al*., 2014).

Another defining feature of grass cell walls is the presence of FA substitution that allows cross‐linking of AX chains or AXs to lignin monomers (Buanafina, 2009; Burr and Fry, 2009). Not surprisingly, an increasing volume of evidence points to the impact of FA‐mediated cross‐linking in saccharification yields as well as in the *in vitro* wall digestibility of grasses (Grabber *et al*., 1998a,b; Iiyama and Lam, 2001; Jung *et al*., 1991; Lam *et al*., 2003). Studies have shown grass‐specific GT61 family members to be involved in mediating such xylan substitutions. Mutants in these genes have little or no arabinofuranose side chains, lower feruloylation and HCAs cross‐linking (Anders *et al*., 2012; Chiniquy *et al*., 2012), in many cases exhibiting increased saccharification, such as *xax1* mutant plants (Chiniquy *et al*., 2012). Even if the pathway for feruloyl esterification is not fully understood, it appears to involve acyltransferases from the BAHD family (Bartley *et al*., 2013). Overexpression of the BAHD acyltransferase *OsAt10* in rice resulted in increased *p*‐CA esterification and reduced FA esterification, and a ~20%–40% increase in saccharification efficiency (Bartley *et al*., 2013). Although the properties of xylan have been changed using transgenic approaches involving GTs (Table 3), one of the potential caveats of overexpressing GTs is that it might lead to saturation of catalytically active GTs in the Golgi apparatus, thereby possibly (i) remodelling xylan formation and/or cross‐linking due to substrate competition and (ii) limiting the availability of other Golgi transmembrane proteins responsible for different xylan substitution patterns.

Despite at least a third of grass cell wall‐related genes having no or few orthologs in *Arabidopsis* (Carpita and McCann, 2008), bioinformatic analysis, transcriptome profiling, and complementation studies using *irx* mutants indicate that several members of the GT43, GT47, and GT61 family have conserved functions in the xylan biosynthetic process across the dicots and monocots (Mitchell *et al*., 2007; Pellny *et al*., 2012). In this context, definitive and direct proof of biochemical function of putative GT43, GT47, GT61, and BAHD grass candidate gene products remain to a greater part unexplored (Table 3). The mechanisms that control the chain length and assembly of the xylan backbone into a functional cell wall are yet unidentified. Discoveries in this research area are appealing and may boost grass cell wall xylan engineering efforts for improved biorefining.

### Mixed‐linkage glucan

Grasses accumulate large amounts (~10%–30%) of non‐branched β‐(1,3;1,4)‐linked glucose residues, also known as mixed‐linkage glucan (MLG), in their primary cell walls (Vogel, 2008). Because of their high and transient accumulation during cell elongation in growing tissues, MLGs have primarily been associated with cell expansion (Carpita and McCann, 2010). However, a higher abundance of MLGs in mature tissues, particularly in the vasculature and sclerenchyma (Vega‐Sánchez *et al*., 2013), and a structural role for MLGs in such tissues (Vega‐Sánchez *et al*., 2012), suggests a broader role for MLG in grasses. The amorphous characteristics of MLG, entirely composed of unbranched and unsubstituted glucose residues yet relatively soluble with low recalcitrance (Burton and Fincher, 2009), make it an attractive target for cell wall engineering aimed at reducing recalcitrance by increasing the amount of easily hydrolysable glucose polymers as well as the ratio of hexose to pentose sugars.

The biosynthesis of MLG involves cellulose synthase‐like proteins CSLF and CSLH (Burton *et al*., 2006; Doblin *et al*., 2009). Recent work has shown that the mutation of a single cellulose synthase‐like gene (*CSLF6*) resulted in a severe reduction or even apparent lack of MLG in rice and barley (Taketa *et al*., 2012; Vega‐Sánchez *et al*., 2012, 2013), demonstrating that *CSLF6* is a dominant gene for controlling the biosynthesis of MLG. Overexpression of the barley *CSLF6* gene under control of the constitutive 35S promoter resulted in a 6‐fold increase of β‐(1,3;1,4) glucans in leaves but also in high mortality as many transgenic barley plants did not survive the transformation process or growth in subsequent generations (Burton *et al*., 2011). This accentuates the need of spatiotemporal regulation when targeting the biosynthesis of MLG. Indeed, heterologous expression of the rice CSLF6 MLG synthase in *Arabidopsis* using a senescence‐associated promoter resulted in up to four times more glucose in the matrix cell wall fraction (without competing with cellulose accumulation) and up to 42% increase in saccharification compared to control lines (Vega‐Sánchez *et al*., 2015) without apparent defects in growth and development. This provides proof of concept that increasing the levels of MLG in grasses when using a promoter that controls the timing of increases in gene expression levels (e.g. employing chemical‐ or temperature‐inducible promoters, or a developmentally regulated promoter), should be feasible. However, as highlighted before, such interventions should be accompanied by careful evaluation of the impact of increasing MLG content on the overall crop fitness. It is also important to highlight that, based on glycome profiling data with the BG1 monoclonal antibody (Meikle *et al*., 1994), some MLGs are firmly integrated into the cell wall matrix as they can only be released after delignification of the cell wall fraction. This has been observed for switchgrass (Shen *et al*., 2013), sugarcane (De Souza *et al*., 2013), maize stover (Li *et al*., 2014), and Miscanthus (da Costa *et al*., 2017), underlining the need to improve our knowledge of the structural associations of MLGs with other cell wall constituents (Kiemle *et al*., 2014; Smith‐Moritz *et al*., 2015) to device engineering strategies based around MLGs.

### Pectin

Pectins are complex, galacturonic acid‐rich, plant cell wall polysaccharides, with homogalacturonan (HG) (~65%) as the most abundant form. For a comprehensive review on the structure and biosynthesis of pectin, we refer to Harholt *et al*. (2010a) and Mohnen (2008). Pectin polysaccharides only constitute a minor component of the cell wall biomass in grasses (~5% of growing cell walls and ~0.1% of mature cell walls (Ishii, 1997)) and have therefore received little attention as a target for optimizing lignocellulosic biomass for biorefining purposes. However, several studies involving ELISA‐based glycome profiling approaches have shown that a proportion of pectin epitopes cannot be released before delignification of the cell wall fraction, including for Miscanthus and switchgrass (da Costa *et al*., 2017; De Souza *et al*., 2015; DeMartini *et al*., 2013; Shen *et al*., 2013), suggesting tight associations between pectin and lignin. It has been postulated that lignin polymerization initiates in the pectin‐rich middle lamella that lies between the walls of adjacent cells and *in vitro* model studies provide evidence that pectin is important for lignin deposition in the cell wall and lignin‐pectin associations can indeed occur (Achyuthan *et al*., 2010; Lairez *et al*., 2005; Wang *et al*., 2013). Additional research is required to address the various hypotheses concerning the exact functional role of pectin during lignification.

One surprising finding was that increasing the ratio of methyl‐esterified pectin to demethyl‐esterified pectin in wheat, through the expression of a kiwifruit pectin methylesterase inhibitor (PMEI), more than doubled saccharification efficiency without adverse effects on plant growth or cell wall deposition (Lionetti *et al*., 2010). PMEIs are inhibitors of pectin methylesterases (PMEs), enzymes that demethyl‐esterify pectins *in muro*, exposing carboxyl residues which can be cross‐linked by calcium (Bosch and Hepler, 2005). Hence, PMEI induced increases in saccharification efficiencies may result from a higher proportion of methyl‐esterified pectins, leading to reduced cell wall cross‐linking and improved accessibility of hydrolytic enzymes to their substrates. Indeed, it appears that the pattern and degree of pectin methyl‐esterification are important in determining the cell wall porosity (Willats *et al*., 2001). It is becoming clear that despite its low content in grass secondary cell walls, pectin polysaccharides can somehow contribute to the cell wall recalcitrance to hydrolysis. Genetic engineering approaches targeting changes in pectin content and/or its substitution pattern might, therefore, provide interesting routes for generating biomass more amenable to saccharification (Latarullo *et al*., 2016). However, more studies are required to establish how pectin modifications affect cell wall recalcitrance in grasses before such approaches can be implemented.

## C. *In planta* production of cell wall degrading or modifying enzymes

The three major cost components associated with the bioconversion of lignocellulosic biomass for use by the biorefining industry are the production of microbial enzymes, feedstocks, and their biochemical processing. The *in planta* production of lignocellulolytic enzymes is a way of tackling all these three important aspects at the same time and has concentrated a lot of research effort. High‐level expression of cell wall degrading (CWD) or modifying enzymes *in planta* is an attractive strategy to alter cell wall architecture, reduce exogenous enzyme production costs, and/or improve plant auto‐hydrolysis for biomass saccharification (Table 4). This approach requires a careful consideration of the strategy for the expression of active enzymes such as the subcellular or tissue targeting, the number of enzymes with different functionalities expressed, and the timing of the expression or activation of the heterologous enzymes.

A range of microbial CWD enzymes including xylanases, cellobiohydrolases (*CBH*) sometimes referred to as exoglucanases (*EXG*), endoglucanases (*ENG*) and β‐glucosidase have been assessed via heterologous production or overexpression in several transgenic grasses, generally yielding no observable negative phenotypic differences and several resulting in enhanced saccharification (Table 4). One iconic example led by the industrial company Agrivida was the expression of an engineered thermostable xylanase gene (*iXynB*) from *Dictyoglomus thermophilum* that remains stable in transgenic maize post‐harvest and only activates upon mild thermochemical pretreatment (Shen *et al*., 2012b). Subsequent enzymatic saccharification of the transgenic plants resulted in ~20% higher glucose and xylose release (Shen *et al*., 2012b). This transgenic modulation demonstrates the feasibility and efficiency of expressing thermostable wall degrading enzymes *in planta* without causing premature auto‐hydrolysis or limiting biomass yield via negative phenotypic impacts. Transgenic rice plants expressing a rice exoglucanase (*EXG1*) under the control of a senescence‐inducible promoter also exhibited ~4%–8% higher saccharification ability of rice straw after senescence and successfully eliminated morphological abnormality or sterility (Furukawa *et al*., 2014), which was observed when *EXG1* was constitutively overexpressed in transgenic rice plants (Nigorikawa *et al*., 2012). In addition to the list of glycosyl hydrolases (Table 4), an *Aspergillus niger* ferulic acid esterase (FAE) has been expressed aimed at altering cell wall composition and reducing recalcitrance during saccharification. The targeted expression of this FAE to the Golgi in *Festuca arundinacea* had no other impact than reduced cell wall ferulates (~14%–25%) and an up to 4% increase in *in vitro* dry matter digestibility on the transgenic plants (Buanafina *et al*., 2010). This effect is likely due to disruption of the ester bonds linking FA to cell wall polysaccharides. For a complete review on *in planta* expression of CWD, please see Furtado *et al*. (2014), Park *et al*. (2016), and Willis *et al*. (2016a).

Although most *in planta* CWD enzyme expression studies have assessed the effect of a single gene encoding for single enzyme activity, complete depolymerisation of lignocellulose requires a suite of CWD enzymes including cellulases, hemicellulases, pectinases, polysaccharide lyases, carbohydrate esterases, laccases, peroxidases, and lytic polysaccharide monooxygenases (LPMOs) with synergistic activities. The principle of producing a cocktail of enzymes as an auto‐hydrolysis system has been applied to tobacco, with the *in planta* production of effective enzymes in the chloroplast that can be used for the generation of fermentable sugars when applied to lignocellulosic biomass (Verma *et al*., 2010). However, there are only a few reports on gene stacking or expression of multiple enzymes aimed at *in planta* hydrolysis. Agrivida employed the co‐expression of an β‐(1,4) endoxylanase with either FAE or an β‐(1,4) endoglucanase to significantly improve hydrolysis (glucose and xylose; and glucose, respectively) of transgenic maize plants compared to controls (Zhang *et al*., 2011), although details about potential effects on plant growth and biomass yield were not reported. An increase in sugar release (31%) was also reported when a FAE was co‐expressed with a senescence‐induced β‐(1,4) endoxylanase in *Festuca arundinacea* but this was accompanied by a 71% decrease in biomass (Buanafina *et al*., 2015). Considerations around the subcellular targeting of CWD enzymes and spatial and temporal control of synthesis and/or activation, coupled with *in planta* expression of multifunctional chimeric genes provide possible routes to mitigate against plant growth issues associated with *in planta* expression of CWD enzymes.

Non‐hydrolytic disruption of lignocellulose (termed amorphogenesis) also provides a viable platform to potentially interfere with cell wall polysaccharide networks and facilitate the accessibility of cellulose to hydrolytic enzymes. Several non‐hydrolytic proteins such as swollenin, carbohydrate binding modules (CBM), loosenin and expansins are thought to induce amorphogenesis through swelling, breaking hydrogen bonding networks and/or pH‐dependent loosening of the cellulose microfibrils or between cellulose and hemicelluloses without lysis of wall polymers (Arantes and Saddler, 2010). Some of these proteins have already been shown to act synergistically when supplemented with hydrolytic enzyme cocktails and to significantly enhance the efficiency of grass cell wall digestibility (Bunterngsook *et al*., 2014; Kim *et al*., 2014; Liu *et al*., 2015). Despite the clear potential of amorphogenesis‐related proteins for improving cellulose accessibility through *in planta* expression, studies, to this end, are merely confined to the expression of plant expansins. The altered expression of endogenous plant expansins *OsEXP4* and *OsEXPA8* in transgenic rice was shown to cause pleiotropic changes in plant growth and development (Choi *et al*., 2003; Ma *et al*., 2013; Wang *et al*., 2014) (Table 4). The authors rationalized this to be a function of altered cell wall compositions, mechanical properties and extensibility from the wall loosening action of expansins. There have been no reports thus far concerning their effect on saccharification and fermentation yields (Table 4).

Recently discovered LPMOs, now classified as auxiliary activity (AA) enzymes in the CAZy database (Levasseur *et al*., 2013), have emerged as key enzymes for the effective degradation of lignocellulosic biomass and have made a significant contribution to the improvement of commercial enzyme cocktails. The two best‐characterized families are AA9 (formerly GH61), mostly fungal enzymes that cleave cellulose chains; and AA10 (formerly CBM33), mostly bacterial enzymes acting on chitin or cellulose. AA9 and AA10 LPMOs share similar 3D structural features and are capable of cleaving polysaccharide chains in their crystalline contexts using an oxidative mechanism that depends on the presence of divalent metal ions and an electron donor (Horn *et al*., 2012; Vaaje‐Kolstad *et al*., 2010). The new chain‐ends generated by LPMOs makes the substrates more susceptible to the activity of glycosyl hydrolases, thus speeding up enzymatic conversion of biomass (Horn *et al*., 2012). Plant cell walls most likely contain sufficient concentrations of electrons delivered by lignin (Dimarogona *et al*., 2012; Westereng *et al*., 2015) and of divalent metal ions (Krzesłowska, 2011) to allow for effective LPMOs activity. Thus, LPMOs could potentially broaden the range of cell wall degrading enzymes for *in planta* expression to facilitate the degradation of cell wall polysaccharides. The identification of new LPMO families and their polysaccharide substrates, which besides cellulose and chitin, now also includes xyloglucan, glucomannan, xylan, MLG, and starch (Hemsworth *et al*., 2015), widens the scope for the oxidative *in planta* ‘pretreatment’ of plant biomass by LPMOs.

## Concluding remarks

The prospect of targeted genetic engineering approaches to improve cell wall biorefining properties of grasses, without significant growth penalties seems complex and challenging. It is important that the research devoted to the biotechnological uses of grasses becomes proportional to their vital significance for the production of food, feed, and materials, as well as feedstock for biorefining. With few exceptions, to date, most genetic engineering approaches to modify cell wall polysaccharides in grasses with the aim of making its biomass more amenable to bioconversion have been fairly crude. Irrespective of the strategy (A, B or C), the development of refined mature genetic engineering approaches in grasses requires (i) a better understanding of grass secondary cell wall biosynthesis, including the roles of the individual cell wall‐associated enzymes and their substrate identities, and the fine cross‐links and structures of secondary cell wall components, and (ii) improved control of the spatiotemporal expression of transgenes encoding enzymes with synergistic or complemental functionalities. With this in mind, rational engineering of cell wall polysaccharides can contribute to an economically sustainable, grass‐derived lignocellulose processing industry.

## References

[pbi12764-bib-0001] Abraham, A. , Mathew, A.K. , Sindhu, R. , Pandey, A. and Binod, P. (2016) Potential of rice straw for bio‐refining: an overview. Bioresour. Technol. 215, 29–36. 27067674 10.1016/j.biortech.2016.04.011

[pbi12764-bib-0002] Abramson, M. , Shoseyov, O. and Shani, Z. (2010) Plant cell wall reconstruction toward improved lignocellulosic production and processability. Plant Sci. 178, 61–72.

[pbi12764-bib-0003] Achyuthan, K.E. , Achyuthan, A.M. , Adams, P.D. , Dirk, S.M. , Harper, J.C. , Simmons, B.A. and Singh, A.K. (2010) Supramolecular self‐assembled chaos: polyphenolic lignin's barrier to cost‐effective lignocellulosic biofuels. Molecules, 15, 8641–8688. 21116223 10.3390/molecules15128641 PMC6259226

[pbi12764-bib-0004] Agarwal, T. , Grotewold, E. , Doseff, A.I. and Gray, J. (2016) MYB31/MYB42 syntelogs exhibit divergent regulation of phenylpropanoid genes in maize, sorghum and rice. Sci. Rep. 6, 28502. 27328708 10.1038/srep28502 PMC4916418

[pbi12764-bib-0005] Agbor, V.B. , Cicek, N. , Sparling, R. , Berlin, A. and Levin, D.B. (2011) Biomass pretreatment: fundamentals toward application. Biotechnol. Adv. 29, 675–685. 21624451 10.1016/j.biotechadv.2011.05.005

[pbi12764-bib-0006] Albersheim, P. , Darvill, A. , Roberts, K. , Sederoff, R. and Staehelin, A. (2011) Plant Cell Walls. New York, NY, USA: Garland Science, Taylor & Francis Group.

[pbi12764-bib-0007] Alvira, P. , Tomás‐Pejó, E. , Ballesteros, M. and Negro, M.J. (2010) Pretreatment technologies for an efficient bioethanol production process based on enzymatic hydrolysis: a review. Bioresour. Technol. 101, 4851–4861. 20042329 10.1016/j.biortech.2009.11.093

[pbi12764-bib-0008] Ambavaram, M.M.R. , Krishnan, A. , Trijatmiko, K.R. and Pereira, A. (2011) Coordinated activation of cellulose and repression of lignin biosynthesis pathways in rice. Plant Physiol. 155, 916–931. 21205614 10.1104/pp.110.168641 PMC3032476

[pbi12764-bib-0009] Anders, N. , Wilkinson, M.D. , Lovegrove, A. , Freeman, J. , Tryfona, T. , Pellny, T.K. , Weimar, T. *et al*. (2012) Glycosyl transferases in family 61 mediate arabinofuranosyl transfer onto xylan in grasses. Proc. Natl Acad. Sci. USA, 109, 989–993. 22215597 10.1073/pnas.1115858109 PMC3271882

[pbi12764-bib-0010] Anderson, W.F. , Sarath, G. , Edme, S. , Casler, M.D. , Mitchell, R.B. , Tobias, C.M. , Hale, A.L. *et al*. (2016) Dedicated herbaceous biomass feedstock genetics and development. Bioenergy Res. 9, 399–411.

[pbi12764-bib-0011] Arantes, V. and Saddler, J.N. (2010) Access to cellulose limits the efficiency of enzymatic hydrolysis: the role of amorphogenesis. Biotechnol. Biofuels, 3, 4. 20178562 10.1186/1754-6834-3-4 PMC2844368

[pbi12764-bib-0012] Balat, M. (2011) Production of bioethanol from lignocellulosic materials via the biochemical pathway: a review. Energy Convers. Manag. 52, 858–875.

[pbi12764-bib-0013] Banerjee, S. (2010) Commercializing lignocellulosic bioethanol: technology bottlenecks and possible remedies. Biofuels Bioprod. Bioref. 4, 77–93.

[pbi12764-bib-0014] Bartley, L.E. , Peck, M.L. , Kim, S.R. , Ebert, B. , Manisseri, C. , Chiniquy, D.M. , Sykes, R. *et al*. (2013) Overexpression of a BAHD acyltransferase, *OsAt10*, alters rice cell wall hydroxycinnamic acid content and saccharification. Plant Physiol. 161, 1615–1633. 23391577 10.1104/pp.112.208694 PMC3613443

[pbi12764-bib-0015] Bhatia, R. and Bosch, M. (2014) Transcriptional regulators of *Arabidopsis* secondary cell wall formation: tools to re‐program and improve cell wall traits. Front. Plant Sci. 5, 192. 24860583 10.3389/fpls.2014.00192 PMC4030196

[pbi12764-bib-0016] Binod, P. , Sindhu, R. , Singhania, R.R. , Vikram, S. , Devi, L. , Nagalakshmi, S. , Kurien, N. *et al*. (2010) Bioethanol production from rice straw: an overview. Bioresour. Technol. 101, 4767–4774. 19944601 10.1016/j.biortech.2009.10.079

[pbi12764-bib-0017] Bischoff, K.P. , Gravois, K.A. , Reagan, T.E. , Hoy, J.W. , Kimbeng, C.A. , LaBorde, C.M. and Hawkins, G.L. (2008) Registration of ‘L 79‐1002’ Sugarcane. J. Plant Regist. 2, 211–217.

[pbi12764-bib-0018] Biswas, G.C.G. , Ransom, C. and Sticklen, M. (2006) Expression of biologically active *acidothermus cellulolyticus* endoglucanase in transgenic maize plants. Plant Sci. 171, 617–623.

[pbi12764-bib-0019] Blümmel, M. , Rao, S.S. , Palaniswami, S. , Shah, L. and Reddy, B.V.S. (2009) Evaluation of sweet sorghum (*Sorghum bicolor L. Moench*) used for bio‐ethanol production in the context of optimizing whole plant utilization. Anim. Nutr. Feed Technol. 9, 1–10.

[pbi12764-bib-0020] Boerjan, W. , Ralph, J. and Baucher, M. (2003) Lignin biosynthesis. Annu. Rev. Plant Biol. 54, 519–546. 14503002 10.1146/annurev.arplant.54.031902.134938

[pbi12764-bib-0021] Bosch, M. and Hepler, P.K. (2005) Pectin methylesterases and pectin dynamics in pollen tubes. Plant Cell, 17, 3219–3226. 16322606 10.1105/tpc.105.037473 PMC1315365

[pbi12764-bib-0022] Bosch, M. , Mayer, C.D. , Cookson, A. and Donnison, I.S. (2011) Identification of genes involved in cell wall biogenesis in grasses by differential gene expression profiling of elongating and non‐elongating maize internodes. J. Exp. Bot. 62, 3545–3561. 21402660 10.1093/jxb/err045 PMC3130177

[pbi12764-bib-0023] Broun, P. (2004) Transcription factors as tools for metabolic engineering in plants. Curr. Opin. Plant Biol. 7, 202–209. 15003222 10.1016/j.pbi.2004.01.013

[pbi12764-bib-0024] Brunecky, R. , Selig, M.J. , Vinzant, T.B. , Himmel, M.E. , Lee, D. , Blaylock, M.J. and Decker, S.R. (2011) *In planta* expression of *A. cellulolyticus* Cel5A endocellulase reduces cell wall recalcitrance in tobacco and maize. Biotechnol. Biofuels, 4, 1. 21269444 10.1186/1754-6834-4-1 PMC3037329

[pbi12764-bib-0025] Brutnell, T.P. , Bennetzen, J.L. and Vogel, J.P. (2015) *Brachypodium distachyon* and *Setaria viridis*: model genetic systems for the grasses. Annu. Rev. Plant Biol. 66, 465–485. 25621515 10.1146/annurev-arplant-042811-105528

[pbi12764-bib-0026] Buanafina, M.M. de O. (2009) Feruloylation in grasses: current and future perspectives. Mol. Plant. 2, 861–872. 19825663 10.1093/mp/ssp067

[pbi12764-bib-0027] Buanafina, M.M. de O. , Langdon, T. , Hauck, B. , Dalton, S.J. and Morris, P. (2006) Manipulating the phenolic acid content and digestibility of Italian ryegrass (*Lolium multiflorum*) by vacuolar‐targeted expression of a fungal ferulic acid esterase. Appl. Biochem. Biotechnol. 129–132, 416–426. 10.1007/978-1-59745-268-7_34 16915658

[pbi12764-bib-0028] Buanafina, M.M. de O. , Langdon, T. , Hauck, B. , Dalton, S. and Morris, P. (2008) Expression of a fungal ferulic acid esterase increases cell wall digestibility of tall fescue (*Festuca arundinacea*). Plant Biotechnol. J. 6, 264–280. 18086237 10.1111/j.1467-7652.2007.00317.x

[pbi12764-bib-0029] Buanafina, M.M. de O. , Langdon, T. , Hauck, B. , Dalton, S. , Timms‐Taravella, E. and Morris, P. (2010) Targeting expression of a fungal ferulic acid esterase to the apoplast, endoplasmic reticulum or golgi can disrupt feruloylation of the growing cell wall and increase the biodegradability of tall fescue (*Festuca arundinacea*). Plant Biotechnol. J. 8, 316–331. 20102533 10.1111/j.1467-7652.2009.00485.x

[pbi12764-bib-0030] Buanafina, M.M. de O. , Langdon, T. , Dalton, S. and Morris, P. (2012) Expression of a *Trichoderma reesei* β‐1,4 endo‐xylanase in tall fescue modifies cell wall structure and digestibility and elicits pathogen defence responses. Planta, 236, 1757–1774. 22878642 10.1007/s00425-012-1724-9

[pbi12764-bib-0031] Buanafina, M.M. de O. , Dalton, S. , Langdon, T. , Timms‐Taravella, E. , Shearer, E.A. and Morris, P. (2015) Functional co‐expression of a fungal ferulic acid esterase and a β‐1,4 endoxylanase in *Festuca arundinacea* (tall fescue) modifies post‐harvest cell wall deconstruction. Planta, 242, 97–111. 25854601 10.1007/s00425-015-2288-2

[pbi12764-bib-0032] Bunterngsook, B. , Mhuantong, W. , Champreda, V. , Thamchaiphenet, A. and Eurwilaichitr, L. (2014) Identification of novel bacterial expansins and their synergistic actions on cellulose degradation. Bioresour. Technol. 159, 64–71. 24632627 10.1016/j.biortech.2014.02.004

[pbi12764-bib-0033] Burr, S.J. and Fry, S.C. (2009) Feruloylated arabinoxylans are oxidatively cross‐linked by extracellular maize peroxidase but not by horseradish peroxidase. Mol. Plant. 2, 883–892. 19825665 10.1093/mp/ssp044

[pbi12764-bib-0034] Burton, R.A. and Fincher, G.B. (2009) (1,3;1,4)‐beta‐D‐glucans in cell walls of the poaceae, lower plants, and fungi: a tale of two linkages. Mol. Plant. 2, 873–882. 19825664 10.1093/mp/ssp063

[pbi12764-bib-0035] Burton, R.A. , Wilson, S.M. , Hrmova, M. , Harvey, A.J. , Shirley, N.J. , Medhurst, A. , Stone, B.A. *et al*. (2006) Cellulose synthase‐like *CslF* genes mediate the synthesis of cell wall (1,3;1,4)‐beta‐D‐glucans. Science, 311, 1940–1942. 16574868 10.1126/science.1122975

[pbi12764-bib-0036] Burton, R.A. , Collins, H.M. , Kibble, N.A.J. , Smith, J.A. , Shirley, N.J. , Jobling, S.A. , Henderson, M. *et al*. (2011) Over‐expression of specific *HvCslF* cellulose synthase‐like genes in transgenic barley increases the levels of cell wall (1,3;1,4)‐β‐d‐glucans and alters their fine structure. Plant Biotechnol. J. 9, 117–135. 20497371 10.1111/j.1467-7652.2010.00532.x

[pbi12764-bib-0037] Byrt, C.S. , Grof, C.P.L. and Furbank, R.T. (2011) C4 plants as biofuel feedstocks: optimising biomass production and feedstock quality from a lignocellulosic perspective. J. Integr. Plant Biol. 53, 120–135. 21205189 10.1111/j.1744-7909.2010.01023.x

[pbi12764-bib-0038] Carpita, N.C. (2012) Progress in the biological synthesis of the plant cell wall: new ideas for improving biomass for bioenergy. Curr. Opin. Biotechnol. 23, 330–337. 22209015 10.1016/j.copbio.2011.12.003

[pbi12764-bib-0039] Carpita, N.C. and McCann, M.C. (2008) Maize and sorghum: genetic resources for bioenergy grasses. Trends Plant Sci. 13, 415–420. 18650120 10.1016/j.tplants.2008.06.002

[pbi12764-bib-0040] Carpita, N.C. and McCann, M.C. (2010) The maize mixed‐linkage (1/3), (1/4)‐β‐D‐glucan polysaccharide is synthesized at the Golgi membrane. Plant Physiol. 153, 1362–1371. 20488897 10.1104/pp.110.156158 PMC2899932

[pbi12764-bib-0041] Carroll, A. and Somerville, C. (2009) Cellulosic biofuels. Annu. Rev. Plant Biol. 60, 165–182. 19014348 10.1146/annurev.arplant.043008.092125

[pbi12764-bib-0042] Cassan‐Wang, H. , Goué, N. , Saidi, M.N. , Legay, S. , Sivadon, P. , Goffner, D. and Grima‐Pettenati, J. (2013) Identification of novel transcription factors regulating secondary cell wall formation in *Arabidopsis* . Front. Plant Sci. 4, 189. 23781226 10.3389/fpls.2013.00189 PMC3677987

[pbi12764-bib-0043] Cesarino, I. , Simões, M.S. , dos Santos Brito, M. , Fanelli, A. , da Franca Silva, T. and Romanel, E. (2016) Building the wall: recent advances in understanding lignin metabolism in grasses. Acta Physiol. Plant. 38, 269.

[pbi12764-bib-0044] Chai, M. , Bellizzi, M. , Wan, C. , Cui, Z. , Li, Y. and Wang, G.L. (2015) The NAC transcription factor OsSWN1 regulates secondary cell wall development in *Oryza sativa* . J. Plant Biol. 58, 44–51.

[pbi12764-bib-0045] Chandel, A.K. , Chandrasekhar, G. , Silva, M.B. and da Silva, S.S. (2012) The realm of cellulases in biorefinery development. Crit. Rev. Biotechnol. 32, 187–202. 21929293 10.3109/07388551.2011.595385

[pbi12764-bib-0046] Chen, X. , Shekiro, J. , Franden, M.A. , Wang, W. , Zhang, M. , Kuhn, E. , Johnson, D.K. *et al*. (2012) The impacts of deacetylation prior to dilute acid pretreatment on the bioethanol process. Biotechnol. Biofuels, 5, 8. 22369467 10.1186/1754-6834-5-8 PMC3309953

[pbi12764-bib-0047] Chiniquy, D. , Sharma, V. , Schultink, A. , Baidoo, E.E. , Rautengarten, C. , Cheng, K. , Carroll, A. *et al*. (2012) XAX1 from glycosyltransferase family 61 mediates xylosyltransfer to rice xylan. Proc. Natl Acad. Sci. USA, 109, 17117–17122. 23027943 10.1073/pnas.1202079109 PMC3479505

[pbi12764-bib-0048] Chiniquy, D. , Varanasi, P. , Oh, T. , Harholt, J. , Katnelson, J. , Singh, S. , Auer, M. *et al*. (2013) Three novel rice genes closely related to the *Arabidopsis IRX9*,* IRX9L*, and *IRX14* genes and their roles in xylan biosynthesis. Front. Plant Sci. 4, 83. 23596448 10.3389/fpls.2013.00083 PMC3622038

[pbi12764-bib-0049] Choi, D. , Lee, Y. , Cho, H.T. and Kende, H. (2003) Regulation of expansin gene expression affects growth and development in transgenic rice plants. Plant Cell, 15, 1386–1398. 12782731 10.1105/tpc.011965 PMC156374

[pbi12764-bib-0050] Chou, H.L. , Dai, Z. , Hsieh, C.W. and Ku, M.S. (2011) High level expression of *Acidothermus cellulolyticus* β‐1, 4‐endoglucanase in transgenic rice enhances the hydrolysis of its straw by cultured cow gastric fluid. Biotechnol. Biofuels, 4, 58. 22152050 10.1186/1754-6834-4-58 PMC3307496

[pbi12764-bib-0051] Chum, H.L. , Warner, E. , Seabra, J.E.A. and Macedo, I.C. (2014) A comparison of commercial ethanol production systems from Brazilian sugarcane and US corn. Biofuels Bioprod. Bioref. 8, 205–223.

[pbi12764-bib-0052] Clifton‐Brown, J. , Hastings, A. , Mos, M. , McCalmont, J.P. , Ashman, C. , Awty‐Carroll, D. , Cerazy, J. *et al*. (2017) Progress in upscaling *Miscanthus* biomass production for the European bio‐ economy with seed based hybrids. GCB Bioenergy, 9, 6–17.

[pbi12764-bib-0053] Cosgrove, D.J. (1997) Assembly and enlargement of the primary cell wall in plants. Annu. Rev. Cell Dev. Biol. 13, 171–201. 9442872 10.1146/annurev.cellbio.13.1.171

[pbi12764-bib-0054] da Costa, R.M.F. , Pattathil, S. , Avci, U. , Lee, S.J. , Hazen, S.P. , Winters, A. , Hahn, M.G. *et al*. (2017) A cell wall reference profile for *Miscanthus* bioenergy crops highlights compositional and structural variations associated with development and organ origin. New Phytol. 213, 1710–1725. 27859277 10.1111/nph.14306 PMC5324610

[pbi12764-bib-0055] De Oliveira, D.M. , Finger‐Teixeira, A. , Rodrigues Mota, T. , Salvador, V.H. , Moreira‐Vilar, F.C. , Correa Molinari, H.B. , Craig Mitchell, R.A. *et al*. (2015) Ferulic acid: a key component in grass lignocellulose recalcitrance to hydrolysis. Plant Biotechnol. J. 13, 1224–1232. 25417596 10.1111/pbi.12292

[pbi12764-bib-0056] De Setta, N. , Monteiro‐Vitorello, C.B. , Metcalfe, C.J. , Cruz, G.M.Q. , Del Bem, L.E. , Vicentini, R. , Nogueira, F.T.S. *et al*. (2014) Building the sugarcane genome for biotechnology and identifying evolutionary trends. BMC Genom. 15, 540. 10.1186/1471-2164-15-540 PMC4122759 24984568

[pbi12764-bib-0057] De Souza, A.P. , Leite, D.C.C. , Pattathil, S. , Hahn, M.G. and Buckeridge, M.S. (2013) Composition and structure of sugarcane cell wall polysaccharides: implications for second‐generation bioethanol production. Bioenergy Res. 6, 564–579.

[pbi12764-bib-0058] De Souza, A.P. , Kamei, C.L.A. , Torres, A.F. , Pattathil, S. , Hahn, M.G. , Trindade, L.M. and Buckeridge, M.S. (2015) How cell wall complexity influences saccharification efficiency in *Miscanthus sinensis* . J. Exp. Bot. 66, 4351–4365. 25908240 10.1093/jxb/erv183 PMC4493786

[pbi12764-bib-0059] DeMartini, J.D. , Pattathil, S. , Miller, J.S. , Li, H. , Hahn, M.G. and Wyman, C.E. (2013) Investigating plant cell wall components that affect biomass recalcitrance in poplar and switchgrass. Energy Environ. Sci. 6, 898–909.

[pbi12764-bib-0060] Dimarogona, M. , Topakas, E. and Christakopoulos, P. (2012) Cellulose degradation by oxidative enzymes. Comput. Struct. Biotechnol. J. 2, e201209015. 24688656 10.5936/csbj.201209015 PMC3962083

[pbi12764-bib-0061] Doblin, M.S. , Pettolino, F.A. , Wilson, S.M. , Campbell, R. , Burton, R.A. , Fincher, G.B. , Newbigin, E. *et al*. (2009) A Barley *cellulose synthase‐like CSLH* gene mediates (1,3; 1,4)‐β‐D‐glucan synthesis in transgenic *Arabidopsis* . Proc. Natl Acad. Sci. USA, 106, 5996–6001. 19321749 10.1073/pnas.0902019106 PMC2667043

[pbi12764-bib-0062] Dong, S. , Delucca, P. , Geijskes, R.J. , Ke, J. , Mayo, K. , Mai, P. , Sainz, M. *et al*. (2014) Advances in *Agrobacterium*‐mediated sugarcane transformation and stable transgene expression. Sugar Tech. 16, 366–371.

[pbi12764-bib-0063] Du, H. , Feng, B.R. , Yang, S.S. , Huang, Y.B. and Tang, Y.X. (2012) The R2R3‐MYB transcription factor gene family in maize. PLoS ONE, 7, e37463. 22719841 10.1371/journal.pone.0037463 PMC3370817

[pbi12764-bib-0064] Dubos, C. , Stracke, R. , Grotewold, E. , Weisshaar, B. , Martin, C. and Lepiniec, L. (2010) MYB transcription factors in *Arabidopsis* . Trends Plant Sci. 15, 573–581. 20674465 10.1016/j.tplants.2010.06.005

[pbi12764-bib-0065] Ebringerová, A. and Heinze, T. (2000) Xylan and xylan derivatives – biopolymers with valuable properties, 1 – Naturally occurring xylans structures, isolation procedures and properties. Macromol. Rapid Commun. 21, 542–556.

[pbi12764-bib-0066] Ebringerová, A. , Hromádková, Z. and Heinze, T. (2005) Hemicellulose. Adv. Polym. Sci. 186, 1–67.

[pbi12764-bib-0067] Eudes, A. , Liang, Y. , Mitra, P. and Loqué, D. (2014) Lignin bioengineering. Curr. Opin. Biotechnol. 26, 189–198. 24607805 10.1016/j.copbio.2014.01.002

[pbi12764-bib-0068] Faik, A. (2010) Xylan biosynthesis: news from the grass. Plant Physiol. 153, 396–402. 20375115 10.1104/pp.110.154237 PMC2879768

[pbi12764-bib-0069] Falter, C. , Zwikowics, C. , Eggert, D. , Blümke, A. , Naumann, M. , Wolff, K. , Ellinger, D. *et al*. (2015) Glucanocellulosic ethanol: the undiscovered biofuel potential in energy crops and marine biomass. Sci. Rep. 5, 13722. 26324382 10.1038/srep13722 PMC4555182

[pbi12764-bib-0070] FAOSTAT (2016) Food Agric. Organ. United Nations Stat. Div. Available at: http://faostat3.fao.org.

[pbi12764-bib-0071] Feltus, F.A. and Vandenbrink, J.P. (2012) Bioenergy grass feedstock: current options and prospects for trait improvement using emerging genetic, genomic, and systems biology toolkits. Biotechnol. Biofuels, 5, 80. 23122416 10.1186/1754-6834-5-80 PMC3502489

[pbi12764-bib-0072] Fornalé, S. , Sonbol, F.M. , Maes, T. , Capellades, M. , Puigdomènech, P. , Rigau, J. and Caparrós‐Ruiz, D. (2006) Down‐regulation of the maize and *Arabidopsis thaliana* caffeic acid *O*‐methyl‐transferase genes by two new maize R2R3‐MYB transcription factors. Plant Mol. Biol. 62, 809–823. 16941210 10.1007/s11103-006-9058-2

[pbi12764-bib-0073] Fornalé, S. , Shi, X. , Chai, C. , Encina, A. , Irar, S. , Capellades, M. , Fuguet, E. *et al*. (2010) ZmMYB31 directly represses maize lignin genes and redirects the phenylpropanoid metabolic flux. Plant J. 64, 633–644. 21070416 10.1111/j.1365-313X.2010.04363.x

[pbi12764-bib-0074] Fouad, W.M. , Hao, W. , Xiong, Y. , Steeves, C. , Sandhu, S.K. and Altpeter, F. (2015) Generation of transgenic energy cane plants with integration of minimal transgene expression cassette. Curr. Pharm. Biotechnol. 16, 407–413. 25751171

[pbi12764-bib-0075] Frame, B. , Main, M. , Schick, R. and Wang, K. (2011) Genetic transformation using maize immature zygotic embryos. Methods Mol. Biol. 710, 327–341. 21207278 10.1007/978-1-61737-988-8_22

[pbi12764-bib-0076] Furtado, A. , Lupoi, J.S. , Hoang, N.V. , Healey, A. , Singh, S. , Simmons, B.A. and Henry, R.J. (2014) Modifying plants for biofuel and biomaterial production. Plant Biotechnol. J. 12, 1246–1258. 25431201 10.1111/pbi.12300

[pbi12764-bib-0077] Furukawa, K. , Ichikawa, S. , Nigorikawa, M. , Sonoki, T. and Ito, Y. (2014) Enhanced production of reducing sugars from transgenic rice expressing exo‐glucanase under the control of a senescence‐inducible promoter. Transgenic Res. 23, 531–537. 24595535 10.1007/s11248-014-9786-z

[pbi12764-bib-0078] Galbe, M. and Zacchi, G. (2012) Pretreatment: the key to efficient utilization of lignocellulosic materials. Biomass Bioenerg. 46, 70–78.

[pbi12764-bib-0079] Grabber, J.H. , Hatfield, R.D. and Ralph, J. (1998a) Diferulate cross‐links impede the enzymatic degradation of non‐lignified maize walls. J. Sci. Food Agric. 77, 193–200.

[pbi12764-bib-0080] Grabber, J.H. , Ralph, J. and Hatfield, R.D. (1998b) Ferulate cross‐links limit the enzymatic degradation of synthetically lignified primary walls of maize. J. Agric. Food Chem. 46, 2609–2614.

[pbi12764-bib-0081] Gray, B.N. , Bougri, O. , Carlson, A.R. , Meissner, J. , Pan, S. , Parker, M.H. , Zhang, D. *et al*. (2011) Global and grain‐specific accumulation of glycoside hydrolase family 10 xylanases in transgenic maize (*Zea mays*). Plant Biotechnol. J. 9, 1100–1108. 21689368 10.1111/j.1467-7652.2011.00632.x

[pbi12764-bib-0082] Grotewold, E. (2008) Transcription factors for predictive plant metabolic engineering: are we there yet? Curr. Opin. Biotechnol. 19, 138–144. 18374558 10.1016/j.copbio.2008.02.002

[pbi12764-bib-0083] Hall, M. , Bansal, P. , Lee, J.H. , Realff, M.J. and Bommarius, A.S. (2010) Cellulose crystallinity ‐ A key predictor of the enzymatic hydrolysis rate. FEBS J. 277, 1571–1582. 20148968 10.1111/j.1742-4658.2010.07585.x

[pbi12764-bib-0084] Handakumbura, P.P. and Hazen, S.P. (2012) Transcriptional regulation of grass secondary cell wall biosynthesis: playing catch‐up with *Arabidopsis thaliana* . Front. Plant Sci. 3, 74. 22639662 10.3389/fpls.2012.00074 PMC3355686

[pbi12764-bib-0085] Harholt, J. , Suttangkakul, A. and Scheller, H.V. (2010a) Biosynthesis of pectin. Plant Physiol. 153, 384–395. 20427466 10.1104/pp.110.156588 PMC2879803

[pbi12764-bib-0086] Harholt, J. , Bach, I.C. , Lind‐Bouquin, S. , Nunan, K.J. , Madrid, S.M. , Brinch‐Pedersen, H. , Holm, P.B. *et al*. (2010b) Generation of transgenic wheat (*Triticum aestivum L*.) accumulating heterologous endo‐xylanase or ferulic acid esterase in the endosperm. Plant Biotechnol. J. 8, 351–362. 20102532 10.1111/j.1467-7652.2009.00490.x

[pbi12764-bib-0087] Harris, D.M. , Corbin, K. , Wang, T. , Gutierrez, R. , Bertolo, A.L. , Petti, C. , Smilgies, D.M. *et al*. (2012) Cellulose microfibril crystallinity is reduced by mutating C‐terminal transmembrane region residues CESA1A903V and CESA3T942I of cellulose synthase. Proc. Natl Acad. Sci. USA, 109, 4098–4103. 22375033 10.1073/pnas.1200352109 PMC3306678

[pbi12764-bib-0088] Harrison, M.D. , Geijskes, J. , Coleman, H.D. , Shand, K. , Kinkema, M. , Palupe, A. , Hassall, R. *et al*. (2011) Accumulation of recombinant cellobiohydrolase and endoglucanase in the leaves of mature transgenic sugar cane. Plant Biotechnol. J. 9, 884–896. 21356003 10.1111/j.1467-7652.2011.00597.x

[pbi12764-bib-0089] Harrison, M.D. , Zhang, Z. , Shand, K. , Chong, B. , Nichols, J. , Oeller, P. , O'Hara, I.M. *et al*. (2014a) The combination of plant‐expressed cellobiohydrolase and low dosages of cellulases for the hydrolysis of sugar cane bagasse. Biotechnol. Biofuels, 7, 131. 25254073 10.1186/s13068-014-0131-9 PMC4172943

[pbi12764-bib-0090] Harrison, M.D. , Geijskes, R.J. , Lloyd, R. , Miles, S. , Palupe, A. , Sainz, M.B. and Dale, J.L. (2014b) Recombinant cellulase accumulation in the leaves of mature, vegetatively propagated transgenic sugarcane. Mol. Biotechnol. 56, 795–802. 24793894 10.1007/s12033-014-9758-9

[pbi12764-bib-0091] Heaton, E. , Voigt, T. and Long, S.P. (2004) A quantitative review comparing the yields of two candidate C_4_ perennial biomass crops in relation to nitrogen, temperature and water. Biomass Bioenerg. 27, 21–30.

[pbi12764-bib-0092] Helle, S. , Cameron, D. , Lam, J. , White, B. and Duff, S. (2003) Effect of inhibitory compounds found in biomass hydrolysates on growth and xylose fermentation by a genetically engineered strain of *S*.* cerevisiae* . Enzyme Microb. Technol. 33, 786–792.

[pbi12764-bib-0093] Hemsworth, G.R. , Johnston, E.M. , Davies, G.J. and Walton, P.H. (2015) Lytic polysaccharide monooxygenases in biomass conversion. Trends Biotechnol. 33, 747–761. 26472212 10.1016/j.tibtech.2015.09.006

[pbi12764-bib-0094] Hirano, K. , Aya, K. , Morinaka, Y. , Nagamatsu, S. , Sato, Y. , Antonio, B.A. , Namiki, N. *et al*. (2013) Survey of genes involved in rice secondary cell wall formation through a co‐expression network. Plant Cell Physiol. 54, 1803–1821. 24089433 10.1093/pcp/pct121

[pbi12764-bib-0095] Hood, E.E. , Love, R. , Lane, J. , Bray, J. , Clough, R. , Pappu, K. , Drees, C. *et al*. (2007) Subcellular targeting is a key condition for high‐level accumulation of cellulase protein in transgenic maize seed. Plant Biotechnol. J. 5, 709–719. 17614952 10.1111/j.1467-7652.2007.00275.x

[pbi12764-bib-0096] Horn, S.J. , Vaaje‐Kolstad, G. , Westereng, B. and Eijsink, V.G. (2012) Novel enzymes for the degradation of cellulose. Biotechnol. Biofuels, 5, 45. 22747961 10.1186/1754-6834-5-45 PMC3492096

[pbi12764-bib-0097] Huang, X. and Wei, Z. (2005) Successful *Agrobacterium*‐mediated genetic transformation of maize elite inbred lines. Plant Cell, Tissue Organ Cult. 83, 187–200.

[pbi12764-bib-0098] Hussey, S.G. , Mizrachi, E. , Creux, N.M. and Myburg, A.A. (2013) Navigating the transcriptional roadmap regulating plant secondary cell wall deposition. Front. Plant Sci. 4, 325. 24009617 10.3389/fpls.2013.00325 PMC3756741

[pbi12764-bib-0099] Hwang, O.J. , Cho, M.A. , Han, Y.J. , Kim, Y.M. , Lim, S.H. , Kim, D.S. , Hwang, I. *et al*. (2014) *Agrobacterium*‐mediated genetic transformation of *Miscanthus sinensis* . Plant Cell, Tissue Organ Cult. 117, 51–63.

[pbi12764-bib-0100] Iiyama, K. and Lam, T.B.T. (2001) Structural characteristics of cell walls of forage grasses – Their nutritional value for ruminant – A review. Asian‐Australasian J. Anim. Sci. 14, 869–879.

[pbi12764-bib-0101] Ishida, Y. , Hiei, Y. and Komari, T. (2007) *Agrobacterium*‐mediated transformation of maize. Nat. Protoc. 2, 1614–1621. 17585302 10.1038/nprot.2007.241

[pbi12764-bib-0102] Ishii, T. (1997) Structure and functions of feruloylated polysaccharides. Plant Sci. 127, 111–127.

[pbi12764-bib-0103] Jönsson, L.J. , Alriksson, B. and Nilvebrant, N.O. (2013) Bioconversion of lignocellulose: inhibitors and detoxification. Biotechnol. Biofuels, 6, 16. 23356676 10.1186/1754-6834-6-16 PMC3574029

[pbi12764-bib-0104] Jung, H.J.G. , Ralph, J. and Hatfield, R.D. (1991) Degradability of phenolic acid‐hemicellulose esters: a model system. J. Sci. Food Agric. 56, 469–478.

[pbi12764-bib-0105] Kiemle, S.N. , Zhang, X. , Esker, A.R. , Toriz, G. , Gatenholm, P. and Cosgrove, D.J. (2014) Role of (1,3)(1,4)‐β‐glucan in cell walls: interaction with cellulose. Biomacromol, 15, 1727–1736. 10.1021/bm5001247 24678830

[pbi12764-bib-0106] Kim, S. and Dale, B.E. (2004) Global potential bioethanol production from wasted crops and crop residues. Biomass Bioenerg. 26, 361–375.

[pbi12764-bib-0107] Kim, W.C. , Ko, J.H. , Kim, J.Y. , Kim, J. , Bae, H.J. and Han, K.H. (2013) MYB46 directly regulates the gene expression of secondary wall‐associated cellulose synthases in Arabidopsis. Plant J. 73, 26–36. 26011122 10.1111/j.1365-313x.2012.05124.x

[pbi12764-bib-0108] Kim, I.J. , Lee, H.J. , Choi, I.G. and Kim, K.H. (2014) Synergistic proteins for the enhanced enzymatic hydrolysis of cellulose by cellulase. Appl. Microbiol. Biotechnol. 98, 8469–8480. 25129610 10.1007/s00253-014-6001-3

[pbi12764-bib-0109] Kimura, T. , Mizutani, T. , Tanaka, T. , Koyama, T. , Sakka, K. and Ohmiya, K. (2003) Molecular breeding of transgenic rice expressing a xylanase domain of the *xynA* gene from *Clostridium thermocellum* . Appl. Microbiol. Biotechnol. 62, 374–379. 12684848 10.1007/s00253-003-1301-z

[pbi12764-bib-0110] Kimura, T. , Mizutani, T. , Sun, J.L. , Kawazu, T. , Karita, S. , Sakka, M. , Kobayashi, Y. *et al*. (2010) Stable production of thermotolerant xylanase B of *Clostridium stercorarium* in transgenic tobacco and rice. Biosci. Biotechnol. Biochem. 74, 954–960. 20460730 10.1271/bbb.90774

[pbi12764-bib-0111] Klein, T.M. , Kornstein, L. , Sanford, J.C. and Fromm, M.E. (1989) Genetic transformation of maize cells by particle bombardment. Plant Physiol. 91, 440–444. 16667039 10.1104/pp.91.1.440 PMC1062012

[pbi12764-bib-0112] Klein‐Marcuschamer, D. , Oleskowicz‐Popiel, P. , Simmons, B.A. and Blanch, H.W. (2012) The challenge of enzyme cost in the production of lignocellulosic biofuels. Biotechnol. Bioeng. 109, 1083–1087. 22095526 10.1002/bit.24370

[pbi12764-bib-0113] Konishi, T. , Aohara, T. , Igasaki, T. , Hayashi, N. , Miyazaki, Y. , Takahashi, A. , Hirochika, H. *et al*. (2011) Down‐regulation of UDP‐arabinopyranose mutase reduces the proportion of arabinofuranose present in rice cell walls. Phytochemistry, 72, 1962–1968. 21824632 10.1016/j.phytochem.2011.07.012

[pbi12764-bib-0114] Krzesłowska, M. (2011) The cell wall in plant cell response to trace metals: polysaccharide remodeling and its role in defense strategy. Acta Physiol. Plant. 33, 35–51.

[pbi12764-bib-0115] Lairez, D. , Cathala, B. , Monties, B. , Bedos‐Belval, F. , Duran, H. and Gorrichon, L. (2005) Aggregation during coniferyl alcohol polymerization in pectin solution: a biomimetic approach of the first steps of lignification. Biomacromol, 6, 763–774. 10.1021/bm049390y 15762640

[pbi12764-bib-0116] Lam, T.B.T. , Kadoya, K. and Iiyama, K. (2001) Bonding of hydroxycinnamic acids to lignin: ferulic and *p*‐coumaric acids are predominantly linked at the benzyl position of lignin, not the β‐position, in grass cell walls. Phytochemistry, 57, 987–992. 11423145 10.1016/s0031-9422(01)00052-8

[pbi12764-bib-0117] Lam, T.B.T. , Iiyama, K. and Stone, B.A. (2003) Hot alkali‐labile linkages in the walls of the forage grass *Phalaris aquatica* and *Lolium perenne* and their relation to in vitro wall digestibility. Phytochemistry, 64, 603–607. 12943783 10.1016/s0031-9422(03)00301-7

[pbi12764-bib-0118] Latarullo, M.B.G. , Tavares, E.Q.P. , Maldonado, G.P. , Leite, D.C.C. and Buckeridge, M.S. (2016) Pectins, endopolygalacturonases, and bioenergy. Front. Plant Sci. 7, 1401. 27703463 10.3389/fpls.2016.01401 PMC5028389

[pbi12764-bib-0119] Lee, C. , Teng, Q. , Zhong, R. , Yuan, Y. and Ye, Z.H. (2014) Functional roles of rice glycosyltransferase family GT43 in xylan biosynthesis. Plant Signal. Behav. 9, e27809. 24525904 10.4161/psb.27809 PMC4091335

[pbi12764-bib-0120] Leon, R.G. , Gilbert, R.A. and Comstock, J.C. (2015) Energycane (*Saccharum spp. × Saccharum spontaneum* L.) biomass production, reproduction, and weed risk assessment scoring in the humid tropics and subtropics. Agron. J. 107, 323–329.

[pbi12764-bib-0121] Levasseur, A. , Drula, E. , Lombard, V. , Coutinho, P.M. and Henrissat, B. (2013) Expansion of the enzymatic repertoire of the CAZy database to integrate auxiliary redox enzymes. Biotechnol. Biofuels, 6, 41. 23514094 10.1186/1754-6834-6-41 PMC3620520

[pbi12764-bib-0122] Li, J. , Ye, X. , An, B. , Du, L. and Xu, H. (2012) Genetic transformation of wheat: current status and future prospects. Plant Biotechnol. Rep. 6, 183–193.

[pbi12764-bib-0123] Li, M. , Pattathil, S. , Hahn, M.G. and Hodge, D.B. (2014) Identification of features associated with plant cell wall recalcitrance to pretreatment by alkaline hydrogen peroxide in diverse bioenergy feedstocks using glycome profiling. R. Soc. Chem. Adv. 4, 17282–17292.

[pbi12764-bib-0124] Lionetti, V. , Francocci, F. , Ferrari, S. , Volpi, C. , Bellincampi, D. , Galletti, R. , D'Ovidio, R. *et al*. (2010) Engineering the cell wall by reducing de‐methyl‐esterified homogalacturonan improves saccharification of plant tissues for bioconversion. Proc. Natl Acad. Sci. USA, 107, 616–621. 20080727 10.1073/pnas.0907549107 PMC2818903

[pbi12764-bib-0125] Liu, G. and Godwin, I.D. (2012) Highly efficient sorghum transformation. Plant Cell Rep. 31, 999–1007. 22234443 10.1007/s00299-011-1218-4 PMC3351618

[pbi12764-bib-0126] Liu, X. , Ma, Y. and Zhang, M. (2015) Research advances in expansins and expansion‐like proteins involved in lignocellulose degradation. Biotechnol. Lett. 37, 1541–1551. 25957563 10.1007/s10529-015-1842-0

[pbi12764-bib-0127] Liu, B. , Gomez, L.D. , Hua, C. , Sun, L. , Ali, I. , Huang, L. , Yu, C. *et al*. (2016) Linkage mapping of stem saccharification digestibility in rice. PLoS ONE, 11, e0159117. 27415441 10.1371/journal.pone.0159117 PMC4944936

[pbi12764-bib-0128] Lovegrove, A. , Wilkinson, M.D. , Freeman, J. , Pellny, T.K. , Tosi, P. , Saulnier, L. , Shewry, P.R. *et al*. (2013) RNA interference suppression of genes in glycosyl transferase families 43 and 47 in wheat starchy endosperm causes large decreases in arabinoxylan content. Plant Physiol. 163, 95–107. 23878080 10.1104/pp.113.222653 PMC3762668

[pbi12764-bib-0129] Ma, Q.‐H. , Wang, C. and Zhu, H.‐H. (2011) TaMYB4 cloned from wheat regulates lignin biosynthesis through negatively controlling the transcripts of both cinnamyl alcohol dehydrogenase and cin‐ namoyl‐CoA reductase genes. Biochimie, 93, 1179–1186. 21536093 10.1016/j.biochi.2011.04.012

[pbi12764-bib-0130] Ma, N. , Wang, Y. , Qiu, S. , Kang, Z. , Che, S. , Wang, G. and Huang, J. (2013) Overexpression of *OsEXPA8*, a root‐specific gene, improves rice growth and root system architecture by facilitating cell extension. PLoS ONE, 8, e75997. 24124527 10.1371/journal.pone.0075997 PMC3790854

[pbi12764-bib-0131] Marriott, P.E. , Gómez, L.D. and McQueen‐Mason, S.J. (2015) Unlocking the potential of lignocellulosic biomass through plant science. New Phytol. 209, 1366–1381. 26443261 10.1111/nph.13684

[pbi12764-bib-0132] Martin, C. , Luo, J. , Lebouteiller, B. , Mock, H.P. , Matros, A. , Peterek, S. , Schijlen, E.G.W.M. *et al*. (2012) Combining genomics and metabolomics for the discovery of regulatory genes and their use in metabolic engineering to produce ‘Healthy Foods’. Acta Hort. 941, 73–84.

[pbi12764-bib-0133] Mayavan, S. , Subramanyam, K. , Jaganath, B. , Sathish, D. , Manickavasagam, M. and Ganapathi, A. (2015) *Agrobacterium*‐mediated in planta genetic transformation of sugarcane setts. Plant Cell Rep. 34, 1835–1848. 26152769 10.1007/s00299-015-1831-8

[pbi12764-bib-0134] Mei, C. , Park, S.H. , Sabzikar, R. , Qi, C. , Ransom, C. and Sticklen, M. (2009) Green tissue‐specific production of a microbial endo‐cellulase in maize (*Zea mays* L.) endoplasmic‐reticulum and mitochondria converts cellulose into fermentable sugars. J. Chem. Technol. Biotechnol. 84, 689–695.

[pbi12764-bib-0135] Meikle, P.J. , Hoogenraad, N.J. , Bonig, I. , Clarke, A.E. and Stone, B.A. (1994) A (1‐3,1‐4)‐β‐glucan‐specific monoclonal antibody and its use in the quantitation and immunocytochemical location of (1‐3,1‐4)‐β‐glucans. Plant J. 5, 1–9. 8130794 10.1046/j.1365-313x.1994.5010001.x

[pbi12764-bib-0136] Merrick, P. and Fei, S. (2015) Plant regeneration and genetic transformation in switchgrass ‐ A review. J. Integr. Agric. 14, 483–493.

[pbi12764-bib-0137] Mitchell, R.A.C. , Dupree, P. and Shewry, P.R. (2007) A novel bioinformatics approach identifies candidate genes for the synthesis and feruloylation of arabinoxylan. Plant Physiol. 144, 43–53. 17351055 10.1104/pp.106.094995 PMC1913792

[pbi12764-bib-0138] Mohnen, D. (2008) Pectin structure and biosynthesis. Curr. Opin. Plant Biol. 11, 266–277. 18486536 10.1016/j.pbi.2008.03.006

[pbi12764-bib-0139] Mottiar, Y. , Vanholme, R. , Boerjan, W. , Ralph, J. and Mansfield, S.D. (2016) Designer lignins: harnessing the plasticity of lignification. Curr. Opin. Biotechnol. 37, 190–200. 26775114 10.1016/j.copbio.2015.10.009

[pbi12764-bib-0140] Nguyen, V.H. , Topno, S. , Balingbing, C. , Nguyen, V.C.N. , Röder, M. , Quilty, J. , Jamieson, C. *et al*. (2016) Generating a positive energy balance from using rice straw for anaerobic digestion. Energy Reports. 2, 117–122.

[pbi12764-bib-0141] Nigorikawa, M. , Watanabe, A. , Furukawa, K. , Sonoki, T. and Ito, Y. (2012) Enhanced saccharification of rice straw by overexpression of rice exo‐glucanase. Rice, 5, 14. 24279714 10.1186/1939-8433-5-14 PMC4883724

[pbi12764-bib-0142] Noda, S. , Koshiba, T. , Hattori, T. , Yamaguchi, M. , Suzuki, S. and Umezawa, T. (2015) The expression of a rice secondary wall‐specific cellulose synthase gene, *OsCesA7*, is directly regulated by a rice transcription factor, OsMYB58/63. Planta, 242, 589–600. 26070439 10.1007/s00425-015-2343-z

[pbi12764-bib-0143] Nordberg, H. , Cantor, M. , Dusheyko, S. , Hua, S. , Poliakov, A. , Shabalov, I. , Smirnova, T. *et al*. (2014) The genome portal of the department of energy joint genome institute: 2014 updates. Nucleic Acids Res. 42, 26–31. 10.1093/nar/gkt1069 PMC3965075 24225321

[pbi12764-bib-0144] Oanh, N.T. , Bich, T.L. , Tipayarom, D. , Manadhar, B.R. , Prapat, P. , Simpson, C.D. and Liu, L.J. (2011) Characterization of particulate matter emission from open burning of rice straw. Atmos. Environ. 45, 493–502. 10.1016/j.atmosenv.2010.09.023 PMC3018782 21243095

[pbi12764-bib-0145] Olsen, A.N. , Ernst, H.A. , Leggio, L.L. and Skriver, K. (2005) NAC transcription factors: structurally distinct, functionally diverse. Trends Plant Sci. 10, 79–87. 15708345 10.1016/j.tplants.2004.12.010

[pbi12764-bib-0146] O'Neill, M.A. and York, W.S. (2003) The composition and structure of plant primary cell walls. In The Plant Cell Wall ( Rose, J.K.C. , ed), pp. 1–54. Boca Raton, FL: CRC Press.

[pbi12764-bib-0147] Oraby, H. , Venkatesh, B. , Dale, B. , Ahmad, R. , Ransom, C. , Oehmke, J. and Sticklen, M. (2007) Enhanced conversion of plant biomass into glucose using transgenic rice‐produced endoglucanase for cellulosic ethanol. Transgenic Res. 16, 739–749. 17237981 10.1007/s11248-006-9064-9

[pbi12764-bib-0148] Park, S.H. , Ransom, C. , Mei, C. , Sabzikar, R. , Qi, C. , Chundawat, S. , Dale, B. *et al*. (2011) The quest for alternatives to microbial cellulase mix production: corn stover‐produced heterologous multi‐cellulases readily deconstruct lignocellulosic biomass into fermentable sugars. J. Chem. Technol. Biotechnol. 86, 633–641.

[pbi12764-bib-0149] Park, S.H. , Ong, R.G. and Sticklen, M. (2016) Strategies for the production of cell wall‐deconstructing enzymes in lignocellulosic biomass and their utilization for biofuel production. Plant Biotechnol. J. 14, 1329–1344. 26627868 10.1111/pbi.12505 PMC5063159

[pbi12764-bib-0150] Patel, M. , Johnson, J.S. , Brettell, R.I.S. , Jacobsen, J. and Xue, G.P. (2000) Transgenic barley expressing a fungal xylanase gene in the endosperm of the developing grains. Mol. Breed. 6, 113–123.

[pbi12764-bib-0151] Paterson, A.H. , Bowers, J.E. , Bruggmann, R. , Dubchak, I. , Grimwood, J. , Gundlach, H. , Haberer, G. *et al*. (2009) The *Sorghum bicolor* genome and the diversification of grasses. Nature, 457, 551–556. 19189423 10.1038/nature07723

[pbi12764-bib-0152] Pauly, M. and Keegstra, K. (2010) Plant cell wall polymers as precursors for biofuels. Curr. Opin. Plant Biol. 13, 304–311. 10.1016/j.pbi.2009.12.009 20097119

[pbi12764-bib-0153] Pauly, M. , Gille, S. , Liu, L. , Mansoori, N. , de Souza, A. , Schultink, A. and Xiong, G. (2013) Hemicellulose biosynthesis. Planta, 238, 627–642. 23801299 10.1007/s00425-013-1921-1

[pbi12764-bib-0154] Pawar, P.M. , Derba‐Maceluch, M. , Chong, S.L. , Gómez, L.D. , Miedes, E. , Banasiak, A. , Ratke, C. *et al*. (2016) Expression of fungal acetyl xylan esterase in *Arabidopsis thaliana* improves saccharification of stem lignocellulose. Plant Biotechnol. J. 14, 387–397. 25960248 10.1111/pbi.12393 PMC11389080

[pbi12764-bib-0155] Pellny, T.K. , Lovegrove, A. , Freeman, J. , Tosi, P. , Love, C.G. , Knox, J.P. , Shewry, P.R. *et al*. (2012) Cell walls of developing wheat starchy endosperm: comparison of composition and RNA‐seq transcriptome. Plant Physiol. 158, 612–627. 22123899 10.1104/pp.111.189191 PMC3271754

[pbi12764-bib-0156] Pereira‐Santana, A. , Alcaraz, L.D. , Castaño, E. , Sanchez‐Calderon, L. , Sanchez‐Teyer, F. and Rodriguez‐Zapata, L. (2015) Comparative genomics of NAC transcriptional factors in angiosperms: implications for the adaptation and diversification of flowering plants. PLoS ONE, 10, e0141866. 26569117 10.1371/journal.pone.0141866 PMC4646352

[pbi12764-bib-0157] Phitsuwan, P. , Sakka, K. and Ratanakhanokchai, K. (2013) Improvement of lignocellulosic biomass *in planta*: a review of feedstocks, biomass recalcitrance, and strategic manipulation of ideal plants designed for ethanol production and processability. Biomass Bioenerg. 58, 390–405.

[pbi12764-bib-0158] Pogorelko, G. , Lionetti, V. , Fursova, O. , Sundaram, R.M. , Qi, M. , Whitham, S.A. , Bogdanove, A.J. *et al*. (2013) Arabidopsis and *Brachypodium distachyon* transgenic plants expressing *Aspergillus nidulans* acetylesterases have decreased degree of polysaccharide acetylation and increased resistance to pathogens. Plant Physiol. 162, 9–23. 23463782 10.1104/pp.113.214460 PMC3641233

[pbi12764-bib-0159] Poovaiah, C.R. , Nageswara‐Rao, M. , Soneji, J.R. , Baxter, H.L. and Stewart, C.N. (2014) Altered lignin biosynthesis using biotechnology to improve lignocellulosic biofuel feedstocks. Plant Biotechnol. J. 12, 1163–1173. 25051990 10.1111/pbi.12225

[pbi12764-bib-0160] Poovaiah, C.R. , Bewg, W.P. , Lan, W. , Ralph, J. and Coleman, H.D. (2016) Sugarcane transgenics expressing MYB transcription factors show improved glucose release. Biotechnol. Biofuels, 9, 143. 27429646 10.1186/s13068-016-0559-1 PMC4946106

[pbi12764-bib-0161] Que, Q. , Elumalai, S. , Li, X. , Zhong, H. , Nalapalli, S. , Schweiner, M. , Fei, X. *et al*. (2014) Maize transformation technology development for commercial event generation. Front. Plant Sci. 5, 379. 25140170 10.3389/fpls.2014.00379 PMC4122164

[pbi12764-bib-0162] Raghuwanshi, A. and Birch, R.G. (2010) Genetic transformation of sweet sorghum. Plant Cell Rep. 29, 997–1005. 20535472 10.1007/s00299-010-0885-x

[pbi12764-bib-0163] Ralph, J. , Bunzel, M. , Marita, J.M. , Hatfield, R.D. , Lu, F. , Kim, H. , Schatz, P.F. *et al*. (2004) Peroxidase‐dependent cross‐linking reactions of *p*‐hydroxycinnamates in plant cell walls. Phytochem. Rev. 3, 79–96.

[pbi12764-bib-0164] Ramamoorthy, R. and Kumar, P.P. (2012) A simplified protocol for genetic transformation of switchgrass (*Panicum virgatum* L.). Plant Cell Rep. 31, 1923–1931. 22733209 10.1007/s00299-012-1305-1

[pbi12764-bib-0165] Ransom, C. , Balan, V. , Biswas, G. , Dale, B. , Crockett, E. and Sticklen, M. (2007) Heterologous *Acidothermus cellulolyticus* 1,4‐β‐endoglucanase E1 produced within the corn biomass converts corn stover into glucose. Appl. Biochem. Biotechnol. 137, 207–219. 18478390 10.1007/s12010-007-9053-3

[pbi12764-bib-0166] Reece‐Hoyes, J.S. and Walhout, A.J. (2012) Gene‐centered yeast one‐hybrid assays. Methods Mol. Biol. 812, 189–208. 22218861 10.1007/978-1-61779-455-1_11 PMC3775493

[pbi12764-bib-0167] Renewable Fuels Association (2017) Available at: http://ethanolrfa.org/wp-content/uploads/2017/02/Ethanol-Industry-Outlook-2017.pdf.

[pbi12764-bib-0168] Rennie, E.A. and Scheller, H.V. (2014) Xylan biosynthesis. Curr. Opin. Biotechnol. 26, 100–107. 24679265 10.1016/j.copbio.2013.11.013

[pbi12764-bib-0169] Rubin, E.M. (2008) Genomics of cellulosic biofuels. Nature, 454, 841–845. 18704079 10.1038/nature07190

[pbi12764-bib-0170] Sah, S.K. , Kaur, A. , Kaur, G. and Singh Cheema, G. (2014) Genetic transformation of rice: problems, progress and prospects. Rice Res. 3, 132.

[pbi12764-bib-0171] Sarkar, N. , Ghosh, S.K. , Bannerjee, S. and Aikat, K. (2012) Bioethanol production from agricultural wastes: an overview. Renew. Energy, 37, 19–27.

[pbi12764-bib-0172] Saulnier, L. , Crépeau, M.J. , Lahaye, M. , Thibault, J.F. , Garcia‐Conesa, M.T. , Kroon, P.A. and Williamson, G. (1999) Isolation and structural determination of two 5,5′‐diferuloyl oligosaccharides indicate that maize heteroxylans are covalently cross‐linked by oxidatively coupled ferulates. Carbohydr. Res. 320, 82–92.

[pbi12764-bib-0173] Scheller, H.V. and Ulvskov, P. (2010) Hemicelluloses. Annu. Rev. Plant Biol. 61, 263–289. 20192742 10.1146/annurev-arplant-042809-112315

[pbi12764-bib-0174] Schultink, A. , Naylor, D. , Dama, M. and Pauly, M. (2015) The role of the plant‐specific ALTERED XYLOGLUCAN9 protein in Arabidopsis cell wall polysaccharide *O*‐acetylation. Plant Physiol. 167, 1271–1283. 25681330 10.1104/pp.114.256479 PMC4378174

[pbi12764-bib-0175] Scully, E.D. , Gries, T. , Sarath, G. , Palmer, N.A. , Baird, L. , Serapiglia, M.J. , Dien, B.S. *et al*. (2016) Overexpression of *SbMyb60* impacts phenylpropanoid biosynthesis and alters secondary cell wall composition in *Sorghum bicolor* . Plant J. 85, 378–395. 26712107 10.1111/tpj.13112

[pbi12764-bib-0176] Selig, M.J. , Adney, W.S. , Himmel, M.E. and Decker, S.R. (2009) The impact of cell wall acetylation on corn stover hydrolysis by cellulolytic and xylanolytic enzymes. Cellulose, 16, 711–722.

[pbi12764-bib-0177] Shen, H. , He, X. , Poovaiah, C.R. , Wuddineh, W.A. , Ma, J. , Mann, D.G.J. , Wang, H. *et al*. (2012a) Functional characterization of the switchgrass (*Panicum virgatum*) R2R3‐MYB transcription factor *PvMYB4* for improvement of lignocellulosic feedstocks. New Phytol. 193, 121–136. 21988539 10.1111/j.1469-8137.2011.03922.x

[pbi12764-bib-0178] Shen, B. , Sun, X. , Zuo, X. , Shilling, T. , Apgar, J. , Ross, M. , Bougri, O. *et al*. (2012b) Engineering a thermoregulated intein‐modified xylanase into maize for consolidated lignocellulosic biomass processing. Nat. Biotechnol. 30, 1131–1136. 23086202 10.1038/nbt.2402

[pbi12764-bib-0179] Shen, H. , Poovaiah, C.R. , Ziebell, A. , Tschaplinski, T.J. , Pattathil, S. , Gjersing, E. , Engle, N.L. *et al*. (2013) Enhanced characteristics of genetically modified switchgrass (*Panicum virgatum* L.) for high biofuel production. Biotechnol. Biofuels, 6, 71. 23651942 10.1186/1754-6834-6-71 PMC3652750

[pbi12764-bib-0180] Sims, R.E.H. , Mabee, W. , Saddler, J.N. and Taylor, M. (2010) An overview of second generation biofuel technologies. Bioresour. Technol. 101, 1570–1580. 19963372 10.1016/j.biortech.2009.11.046

[pbi12764-bib-0181] Slavov, G. , Allison, G. and Bosch, M. (2013) Advances in the genetic dissection of plant cell walls: tools and resources available in *Miscanthus* . Front. Plant Sci. 4, 217. 23847628 10.3389/fpls.2013.00217 PMC3701120

[pbi12764-bib-0182] Slavov, G.T. , Nipper, R. , Robson, P. , Farrar, K. , Allison, G.G. , Bosch, M. , Clifton‐Brown, J.C. *et al*. (2014) Genome‐wide association studies and prediction of 17 traits related to phenology, biomass and cell wall composition in the energy grass *Miscanthus sinensis* . New Phytol. 201, 1227–1239. 24308815 10.1111/nph.12621 PMC4284002

[pbi12764-bib-0183] Smith‐Moritz, A.M. , Hao, Z. , Fernández‐Niño, S.G. , Fangel, J.U. , Verhertbruggen, Y. , Holman, H.Y.N. , Willats, W.G.T. *et al*. (2015) Structural characterization of a mixed‐linkage glucan deficient mutant reveals alteration in cellulose microfibril orientation in rice coleoptile mesophyll cell walls. Front. Plant Sci. 6, 628. 26347754 10.3389/fpls.2015.00628 PMC4539472

[pbi12764-bib-0184] Sonbol, F.M. , Fornalé, S. , Capellades, M. , Encina, A. , Touriño, S. , Torres, J.L. , Rovira, P. *et al*. (2009) The maize *Zm*MYB42 represses the phenylpropanoid pathway and affects the cell wall structure, composition and degradability in *Arabidopsis thaliana* . Plant Mol. Biol. 70, 283–296. 19238561 10.1007/s11103-009-9473-2

[pbi12764-bib-0185] Song, X.Q. , Liu, L.F. , Jiang, Y.J. , Zhang, B.C. , Gao, Y.P. , Liu, X.L. , Lin, Q.S. *et al*. (2013) Disruption of secondary wall cellulose biosynthesis alters cadmium translocation and tolerance in rice plants. Mol. Plant. 6, 768–780. 23376772 10.1093/mp/sst025

[pbi12764-bib-0186] Souza, G.M. , Berges, H. , Bocs, S. , Casu, R. , D'Hont, A. , Ferreira, J.E. , Henry, R. *et al*. (2011) The sugarcane genome challenge: strategies for sequencing a highly complex genome. Trop. Plant Biol. 4, 145–156.

[pbi12764-bib-0187] Sparks, C.A. , Doherty, A. and Jones, H.D. (2014) Genetic transformation of wheat via *Agrobacterium*‐mediated DNA delivery. Methods Mol. Biol. 1099, 235–250. 24243208 10.1007/978-1-62703-715-0_19

[pbi12764-bib-0188] Sumiyoshi, M. , Nakamura, A. , Nakamura, H. , Hakata, M. , Ichikawa, H. , Hirochika, H. , Ishii, T. *et al*. (2013) Increase in cellulose accumulation and improvement of saccharification by overexpression of arabinofuranosidase in rice. PLoS ONE, 8, e78269. 24223786 10.1371/journal.pone.0078269 PMC3817243

[pbi12764-bib-0189] Swaminathan, K. , Alabady, M.S. , Varala, K. , De Paoli, E. , Ho, I. , Rokhsar, D.S. , Arumuganathan, A.K. *et al*. (2010) Genomic and small RNA sequencing of *Miscanthus x giganteus* shows the utility of sorghum as a reference genome sequence for Andropogoneae grasses. Genome Biol. 11, R12. 20128909 10.1186/gb-2010-11-2-r12 PMC2872872

[pbi12764-bib-0190] Taketa, S. , Yuo, T. , Tonooka, T. , Tsumuraya, Y. , Inagaki, Y. , Haruyama, N. , Larroque, O. *et al*. (2012) Functional characterization of barley betaglucanless mutants demonstrates a unique role for CslF6 in (1,3;1,4)‐β‐D‐glucan biosynthesis. J. Exp. Bot. 63, 381–392. 21940720 10.1093/jxb/err285 PMC3245474

[pbi12764-bib-0191] Tan, H.T. , Shirley, N.J. , Singh, R.R. , Henderson, M. , Dhugga, K.S. , Mayo, G.M. , Fincher, G.B. *et al*. (2015) Powerful regulatory systems and post‐transcriptional gene silencing resist increases in cellulose content in cell walls of barley. BMC Plant Biol. 15, 62. 25850007 10.1186/s12870-015-0448-y PMC4349714

[pbi12764-bib-0192] Tanaka, K. , Murata, K. , Yamazaki, M. , Onosato, K. , Miyao, A. and Hirochika, H. (2003) Three distinct rice cellulose synthase catalytic subunit genes required for cellulose synthesis in the secondary wall. Plant Physiol. 133, 73–83. 12970476 10.1104/pp.103.022442 PMC196581

[pbi12764-bib-0193] Taylor‐Teeples, M. , Lin, L. , de Lucas, M. , Turco, G. , Toal, T.W. , Gaudinier, A. , Young, N.F. *et al*. (2015) An *Arabidopsis* gene regulatory network for secondary cell wall synthesis. Nature, 517, 571–575. 25533953 10.1038/nature14099 PMC4333722

[pbi12764-bib-0194] Turner, S.R. and Somerville, C.R. (1997) Collapsed xylem phenotype of Arabidopsis identifies mutants deficient in cellulose deposition in the secondary cell wall. Plant Cell, 9, 689–701. 9165747 10.1105/tpc.9.5.689 PMC156949

[pbi12764-bib-0195] Vaaje‐Kolstad, G. , Westereng, B. , Horn, S.J. , Liu, Z. , Zhai, H. , Sørlie, M. and Eijsink, V.G.H. (2010) An oxidative enzyme boosting the enzymatic conversion of recalcitrant polysaccharides. Science, 330, 219–222. 20929773 10.1126/science.1192231

[pbi12764-bib-0196] Valdivia, E.R. , Herrera, M.T. , Gianzo, C. , Fidalgo, J. , Revilla, G. , Zarra, I. and Sampedro, J. (2013) Regulation of secondary wall synthesis and cell death by NAC transcription factors in the monocot *Brachypodium distachyon* . J. Exp. Bot. 64, 1333–1343. 23386682 10.1093/jxb/ers394 PMC3598421

[pbi12764-bib-0197] Van der Weijde, T. , Alvim Kamei, C.L. , Torres, A.F. , Vermerris, W. , Dolstra, O. , Visser, R.G. and Trindade, L.M. (2013) The potential of C4 grasses for cellulosic biofuel production. Front. Plant Sci. 4, 107. 23653628 10.3389/fpls.2013.00107 PMC3642498

[pbi12764-bib-0198] Vega‐Sánchez, M.E. , Verhertbruggen, Y. , Christensen, U. , Chen, X. , Sharma, V. , Varanasi, P. , Jobling, S.A. *et al*. (2012) Loss of *Cellulose synthase‐like F6* function affects mixed‐linkage glucan deposition, cell wall mechanical properties, and defense responses in vegetative tissues of rice. Plant Physiol. 159, 56–69. 22388489 10.1104/pp.112.195495 PMC3375985

[pbi12764-bib-0199] Vega‐Sánchez, M.E. , Verhertbruggen, Y. , Scheller, H.V. and Ronald, P.C. (2013) Abundance of mixed linkage glucan in mature tissues and secondary cell walls of grasses. Plant Signal. Behav. 8, e23143. 23299432 10.4161/psb.23143 PMC3657012

[pbi12764-bib-0200] Vega‐Sánchez, M.E. , Loqué, D. , Lao, J. , Catena, M. , Verhertbruggen, Y. , Herter, T. , Yang, F. *et al*. (2015) Engineering temporal accumulation of a low recalcitrance polysaccharide leads to increased C6 sugar content in plant cell walls. Plant Biotechnol. J. 13, 903–914. 25586315 10.1111/pbi.12326

[pbi12764-bib-0201] Verma, D. , Kanagaraj, A. , Jin, S. , Singh, N.D. , Kolattukudy, P.E. and Daniell, H. (2010) Chloroplast‐derived enzyme cocktails hydrolyse lignocellulosic biomass and release fermentable sugars. Plant Biotechnol. J. 8, 332–350. 20070870 10.1111/j.1467-7652.2009.00486.x PMC2854225

[pbi12764-bib-0202] Vogel, J. (2008) Unique aspects of the grass cell wall. Curr. Opin. Plant Biol. 11, 301–307. 18434239 10.1016/j.pbi.2008.03.002

[pbi12764-bib-0203] Wang, X. , Yamada, T. , Kong, F.J. , Abe, Y. , Hoshino, Y. , Sato, H. , Takamizo, T. *et al*. (2011) Establishment of an efficient *in vitro* culture and particle bombardment‐mediated transformation systems in *Miscanthus sinensis* Anderss., a potential bioenergy crop. GCB Bioenergy, 3, 322–332.

[pbi12764-bib-0204] Wang, P. , Fan, J. and Xie, Y. (2013) Synthesis and characterization of pectin‐dehydrogenation polymer complex by isotopic labeling method. Cellul. Chem. Technol. 47, 401–408.

[pbi12764-bib-0205] Wang, Y. , Ma, N. , Qiu, S. , Zou, H. , Zang, G. , Kang, Z. , Wang, G. *et al*. (2014) Regulation of the α‐expansin gene *OsEXPA8* expression affects root system architecture in transgenic rice plants. Mol. Breed. 34, 47–57.

[pbi12764-bib-0206] Wang, X. , Tang, Q. , Zhao, X. , Jia, C. , Yang, X. , He, G. , Wu, A. *et al*. (2016) Functional conservation and divergence of *Miscanthus lutarioriparius* GT43 gene family in xylan biosynthesis. BMC Plant Biol. 16, 102. 27114083 10.1186/s12870-016-0793-5 PMC4845329

[pbi12764-bib-0207] Weng, X. , Huang, Y. , Hou, C. and Jiang, D. (2013) Effects of an exogenous xylanase gene expression on the growth of transgenic rice and the expression level of endogenous xylanase inhibitor gene *RIXI* . J. Sci. Food Agric. 93, 173–179. 22674383 10.1002/jsfa.5746

[pbi12764-bib-0208] Westereng, B. , Cannella, D. , Wittrup Agger, J. , Jørgensen, H. , Larsen Andersen, M. , Eijsink, V.G.H. and Felby, C. (2015) Enzymatic cellulose oxidation is linked to lignin by long‐range electron transfer. Sci. Rep. 5, 18561. 26686263 10.1038/srep18561 PMC4685257

[pbi12764-bib-0209] Willats, W.G.T. , Mccartney, L. , Mackie, W. and Knox, J.P. (2001) Pectin: cell biology and prospects for functional analysis. Plant Mol. Biol. 47, 9–27. 11554482

[pbi12764-bib-0210] Willis, J.D. , Mazarei, M. and Stewart, C.N. (2016a) Transgenic plant‐produced hydrolytic enzymes and the potential of insect gut‐derived hydrolases for biofuels. Front. Plant Sci. 7, 675. 27303411 10.3389/fpls.2016.00675 PMC4885837

[pbi12764-bib-0211] Willis, J.D. , Smith, J.A. , Mazarei, M. , Zhang, J.Y. , Turner, G.B. , Decker, S.R. , Sykes, R.W. *et al*. (2016b) Downregulation of a UDP‐arabinomutase gene in switchgrass (*Panicum virgatum* L.) results in increased cell wall lignin while reducing arabinose‐glycans. Front Plant Sci. 7, 1580. 27833622 10.3389/fpls.2016.01580 PMC5081414

[pbi12764-bib-0212] Wu, H. and Altpeter, F. (2015) Sugarcane (*Saccharum* Spp. hybrids). Methods Mol. Biol. 1224, 307–316. 25416267 10.1007/978-1-4939-1658-0_24

[pbi12764-bib-0213] Wuddineh, W.A. , Mazarei, M. , Turner, G.B. , Sykes, R.W. , Decker, S.R. , Davis, M.F. and Stewart, C.N. (2015) Identification and molecular characterization of the switchgrass AP2/ERF transcription factor superfamily, and overexpression of *PvERF001* for improvement of biomass characteristics for biofuel. Front. Bioeng. Biotechnol. 3, 101. 26258121 10.3389/fbioe.2015.00101 PMC4507462

[pbi12764-bib-0214] Wuddineh, W.A. , Mazarei, M. , Zhang, J.Y. , Turner, G.B. , Sykes, R.W. , Decker, S.R. , Davis, M.F. *et al*. (2016) Identification and overexpression of a Knotted1‐like transcription factor in switchgrass (*Panicum virgatum* L.) for lignocellulosic feedstock improvement. Front Plant Sci. 7, 520. 27200006 10.3389/fpls.2016.00520 PMC4848298

[pbi12764-bib-0215] Xi, Y. , Ge, Y. and Wang, Z.Y. (2009) Genetic transformation of switchgrass. Methods Mol. Biol. 581, 53–59. 19768615 10.1007/978-1-60761-214-8_4

[pbi12764-bib-0216] Xu, X. , Zhang, Y. , Meng, Q. , Meng, K. , Zhang, W. , Zhou, X. , Luo, H. *et al*. (2013) Overexpression of a fungal β‐mannanase from *Bispora* sp. MEY‐1 in maize seeds and enzyme characterization. PLoS ONE, 8, e56146. 23409143 10.1371/journal.pone.0056146 PMC3569411

[pbi12764-bib-0217] Xue, G.P. , Patel, M. , Johnson, J.S. , Smyth, D.J. and Vickers, C.E. (2003) Selectable marker‐free transgenic barley producing a high level of cellulase (1,4‐beta‐glucanase) in developing grains. Plant Cell Rep. 21, 1088–1094. 12836003 10.1007/s00299-003-0627-4

[pbi12764-bib-0218] Yang, C. , Li, D. , Liu, X. , Ji, C. , Hao, L. , Zhao, X. , Li, X. *et al*. (2014) OsMYB103L, an R2R3‐MYB transcription factor, influences leaf rolling and mechanical strength in rice (*Oryza sativa* L.). BMC Plant Biol. 14, 158. 24906444 10.1186/1471-2229-14-158 PMC4062502

[pbi12764-bib-0219] Yang, W. , Zhang, Y. , Zhou, X. , Zhang, W. , Xu, X. , Chen, R. , Meng, Q. *et al*. (2015) Production of a highly protease‐resistant fungal α‐galactosidase in transgenic maize seeds for simplified feed processing. PLoS ONE, 10, e0129294. 26053048 10.1371/journal.pone.0129294 PMC4460051

[pbi12764-bib-0220] Yoshida, K. , Sakamoto, S. , Kawai, T. , Kobayashi, Y. , Sato, K. , Ichinose, Y. , Yaoi, K. *et al*. (2013) Engineering the *Oryza sativa* cell wall with rice NAC transcription factors regulating secondary wall formation. Front. Plant Sci. 4, 383. 24098302 10.3389/fpls.2013.00383 PMC3787547

[pbi12764-bib-0221] Yuan, Y. , Teng, Q. , Zhong, R. and Ye, Z.H. (2016) Roles of Arabidopsis TBL34 and TBL35 in xylan acetylation and plant growth. Plant Sci. 243, 120–130. 26795157 10.1016/j.plantsci.2015.12.007

[pbi12764-bib-0222] Zhang, J.Z. (2003) Overexpression analysis of plant transcription factors. Curr. Opin. Plant Biol. 6, 430–440. 12972043 10.1016/s1369-5266(03)00081-5

[pbi12764-bib-0223] Zhang, D. , VanFossen, A.L. , Pagano, R.M. , Johnson, J.S. , Parker, M.H. , Pan, S. , Gray, B.N. *et al*. (2011) Consolidated pretreatment and hydrolysis of plant biomass expressing cell wall degrading enzymes. Bioenergy Res. 4, 276–286.

[pbi12764-bib-0224] Zhang, Q. , Zhang, W. , Lin, C. , Xu, X. and Shen, Z. (2012) Expression of an *Acidothermus cellulolyticus* endoglucanase in transgenic rice seeds. Protein Expr. Purif. 82, 279–283. 22306743 10.1016/j.pep.2012.01.011

[pbi12764-bib-0225] Zhang, Y. , Xu, X. , Zhou, X. , Chen, R. , Yang, P. , Meng, Q. , Meng, K. *et al*. (2013) Overexpression of an acidic endo‐β‐1,3‐1,4‐glucanase in transgenic maize seed for direct utilization in animal feed. PLoS ONE, 8, e81993. 24391711 10.1371/journal.pone.0081993 PMC3876984

[pbi12764-bib-0226] Zhang, B. , Zhao, T. , Yu, W. , Kuang, B. , Yao, Y. , Liu, T. , Chen, X. *et al*. (2014) Functional conservation of the glycosyltransferase gene *GT47A* in the monocot rice. J. Plant. Res. 127, 423–432. 24723033 10.1007/s10265-014-0631-5

[pbi12764-bib-0227] Zhang, J. , Zhang, S. , Li, H. , Du, H. , Huang, H. , Li, Y. , Hu, Y. *et al*. (2016) Identification of transcription factors ZmMYB111 and ZmMYB148 involved in phenylpropanoid metabolism. Front. Plant Sci. 7, 148. 26913047 10.3389/fpls.2016.00148 PMC4753300

[pbi12764-bib-0228] Zhong, R. , Lee, C. and Ye, Z.H. (2010) Global analysis of direct targets of secondary wall NAC master switches in *Arabidopsis* . Mol Plant. 3, 1087–1103. 20935069 10.1093/mp/ssq062

[pbi12764-bib-0229] Zhong, R. , Lee, C. , McCarthy, R.L. , Reeves, C.K. , Jones, E.G. and Ye, Z.H. (2011) Transcriptional activation of secondary wall biosynthesis by rice and maize NAC and MYB transcription factors. Plant Cell Physiol. 52, 1856–1871. 21908441 10.1093/pcp/pcr123

[pbi12764-bib-0230] Zhong, R. , Yuan, Y. , Spiekerman, J.J. , Guley, J.T. , Egbosiuba, J.C. and Ye, Z.H. (2015) Functional characterization of NAC and MYB transcription factors involved in regulation of biomass production in switchgrass (*Panicum virgatum*). PLoS ONE, 10, e0134611. 26248336 10.1371/journal.pone.0134611 PMC4527753

[pbi12764-bib-0231] Zhou, J. , Lee, C. , Zhong, R. and Ye, Z.H. (2009) MYB58 and MYB63 are transcriptional activators of the lignin biosynthetic pathway during secondary cell wall formation in *Arabidopsis* . Plant Cell, 21, 248–266. 19122102 10.1105/tpc.108.063321 PMC2648072

[pbi12764-bib-0232] Zhu, J.Y. , Sun, Y. and Wang, Z.Y. (2012) Genome‐wide identification of transcription factor‐binding sites in plants using chromatin immunoprecipitation followed by microarray (ChIP‐chip) or sequencing (ChIP‐seq). Methods Mol. Biol. 876, 173–188. 22576095 10.1007/978-1-61779-809-2_14

